# Supplementary material for: Effect of chemical modification on the exon-skipping activity of heteroduplex oligonucleotides
Source: Mol Ther Nucleic Acids. 2025 Feb 1;36(1):102468. doi: 10.1016/j.omtn.2025.102468 (PMC11875208; doi:10.1016/j.omtn.2025.102468)
Supplement: Document S1. Figures S1–S6 and Tables S1–S8 [file mmc1.pdf]

OMTN, Volume *36*

Supplemental information

Effect of chemical modification on the exon-skipping activity of heteroduplex oligonucleotides

Takenori Shimo, Juri Hasegawa, Kotaro Yoshioka, Yusuke Nakatsuji, Kotomi Aso, Keisuke Tachibana, Tetsuya Nagata, Takanori Yokota, and Satoshi Obika

Table S1 Complementary oligonucleotides used for Figure 1.
Complementary oligonucleotides for *DMD* gene exon 58 skipping are shown. Sequences are shown from 5′ to 3′. Uppercase letters: RNA. Lowercase letters: DNA. Uppercase letters with bold and italic: 2′-OMe RNA. ^: phosphorothioate.

Entry	Name	Sequence
01	Wing	***G***^***G***^CCUUCAAGAGG***G***^***A***
02	DNA	ggccttcaagaggga
03	RNA	GGCCUUCAAGAGGGA

Table S2 SSOs used for Figures 1 and 2.

LNA-based SSOs and 2'-OMe RNA based SSOs for *DMD* gene exon 58 skipping are shown. Sequences are shown from 5′ to 3′. Lowercase letter: DNA, uppercase letter with bold and underline: LNA, **C**: LNA-5-methylcytosine, uppercase letters with bold and italic: 2′-OMe RNA, and ^: phosphorothioate.

Entry	Name	Sequence
01	LNA based SSO	t^c^**C**^c^t^**C**^t^t^**G**^a^a^**G**^g^c^**C**
02	2'-OMe based SSO	***U^C^C^C^U^C^U^U^G^A^A^G^G^C^C***

Table S3 Complementary oligonucleotides used for FigureS1.
Complementary oligonucleotides for *DMD* gene exon 58 skipping are shown. Sequences are shown from 5′ to 3′. Uppercase letters: RNA. Lowercase letters: DNA. Uppercase letters with bold and italic: 2′-OMe RNA. ^: phosphorothioate.

Entry	Name	Sequence
01	Wing	***C***^***C***^AGGAGCCCAGA***G***^***G***
02	DNA	Ccaggagcccagagg
03	RNA	CCAGGAGCCCAGAGG

Table S4 SSOs used for FigureS1.

LNA-based SSOs and 2'-OMe RNA based SSOs for *DMD* gene exon 58 skipping are shown. Sequences are shown from 5′ to 3′. Lowercase letter: DNA, uppercase letter with bold and underline: LNA, **C**: LNA-5-methylcytosine, uppercase letters with bold and italic: 2′-OMe RNA, and ^: phosphorothioate.

Entry	Name	Sequence
01	LNA based SSO	**C**^c^t^**C**^t^g^**G**^g^c^**T**^c^c^**T**^g^g
02	2'-OMe based SSO	***C***^***C***^***U***^***C***^***U***^***G***^***G***^***G***^***C***^***U***^***C***^***C***^***U***^***G***^***G***

Table S5 Complementary oligonucleotides used for the assay.
Complementary oligonucleotides for *DMD* gene exon 58 skipping are shown. Sequences are shown from 5′ to 3′. Lowercase letter: DNA, uppercase letter: RNA, uppercase letter with bold and underline: LNA, uppercase letters with bold and italic: 2′-OMe RNA, i: 2′-deoxyinosine, I: inosine, and ^: phosphorothioate.

Entry	Name	Sequence
1	D1	acaggccttcaagagggaatt
2	D2	ggccttcaagaggga
	(also called "DNA" in Fig.1)	
3	D3	cttcaagag
4	D4	g^g^c^c^t^t^c^a^a^g^a^g^g^g^a
5	D5	g^g^ccttcaagaggg^a
6	D6	<u>**G**</u>^<u>**G**</u>^ccttcaagagg<u>**G**</u>^<u>**A**</u>
7	D7	iiccttcaaiaiiia
8	R1	ACAGGCCUUCAAGAGGGAAUU
9	R2	GGCCUUCAAGAGGGA
	(also called "RNA" in Fig.1)	
10	R3	CUUCAAGAG
11	R4	G^G^C^C^U^U^C^A^A^G^A^G^G^G^A
12	R5	G^G^CCUUCAAGAGGG^A
13	R6	<u>**G**</u>^<u>**G**</u>^CCUUCAAGAGG<u>**G**</u>^<u>**A**</u>
14	R7	IICCUUCAAIAIIIA
15	Wing	***G***^***G***^CCUUCAAGAGG***G***^***A***

Table S6 SSOs and complementary oligonucleotides used for the time-lapse microscopy imaging.

LNA-based SSOs and 2'-OMe RNA based SSOs for *DMD* gene exon 58 skipping and their complementary oligonucleotides are shown. Sequences are shown from 5′ to 3′. Lowercase letter: DNA, uppercase letter: RNA, uppercase letter with bold and underline: LNA, **C**: LNA-5-methylcytosine, uppercase letters with bold and italic: 2′-OMe RNA, f: 6-FAM, Y_NHC6: Alexa-647 with an aminohexyl linker and ^: phosphorothioate.

Entry	Name	Sequence
01	f_DMDexon58-1+14_5/15	f^t^c^**C**^c^t^**C**^t^t^**G**^a^a^**G**^g^c^**C**
02	Y_wing	Y_NHC6^***G***^***G***^CCUUCAAGAGG***G***^***A***
03	Y_R2	Y_NHC6^GGCCUUCAAGAGGGA
04	Y_R4	Y_NHC6^G^G^C^C^U^U^C^A^A^G^A^G^G^G^A
05	Y_R5	Y_NHC6^G^G^CCUUCAAGAGGG^A
06	Y_R6	Y_NHC6^**G**^**G**^CCUUCAAGAGG**G**^**A**

Table S7 SSOs and complementary oligonucleotides used for murine study.
LNA-based SSO for murine *Dmd* gene exon 23 skipping and its complementary oligonucleotides are shown. Sequences are shown from 5′ to 3′. Lowercase letter: DNA, uppercase letter: RNA, uppercase letter with bold and underline: LNA, <u>**C**</u>: LNA-5-methylcytosine, Toc_: tocopherol and ^: phosphorothioate.

Entry	Name	Sequence
01	LNA based SSO	a^<u>**C**</u>^c^<u>**T**</u>^c^<u>**G**</u>^g^<u>**C**</u>^t^<u>**T**</u>^a^<u>**C**</u>^c
02	Comp. RNA	G^G^U^AAGCCGA^G^G^U
03	Toc_Comp. RNA	Toc_G^G^U^AAGCCGA^G^G^U

Table S8 DNA primers used for evaluation of exon skipping efficiencies in stable cell line. DNA primers for RT-qPCR are shown. Sequences are shown from 5′ to 3′. Uppercase letters: DNA.

Entry	Gene		Sequence	Product size
01	*DMD exon 58*	Fw	AGTTCTGACCAGTGGAAGCG	156 bp
02	*skipping*	Rv	CCTCAGGAGGCAGCTCCTAT	
03	*GAPDH*	Fw	GAGTCAACGGATTTGGTCGT	185 bp
04		Rv	GACAAGCTTCCCGTTCTCAG	
05	*RPLP2*	Fw	TGGACAGCGTGGGTATCGAG	92 bp
06		Rv	CTGGGCAATGACGTCTTCAA	

FigureS1

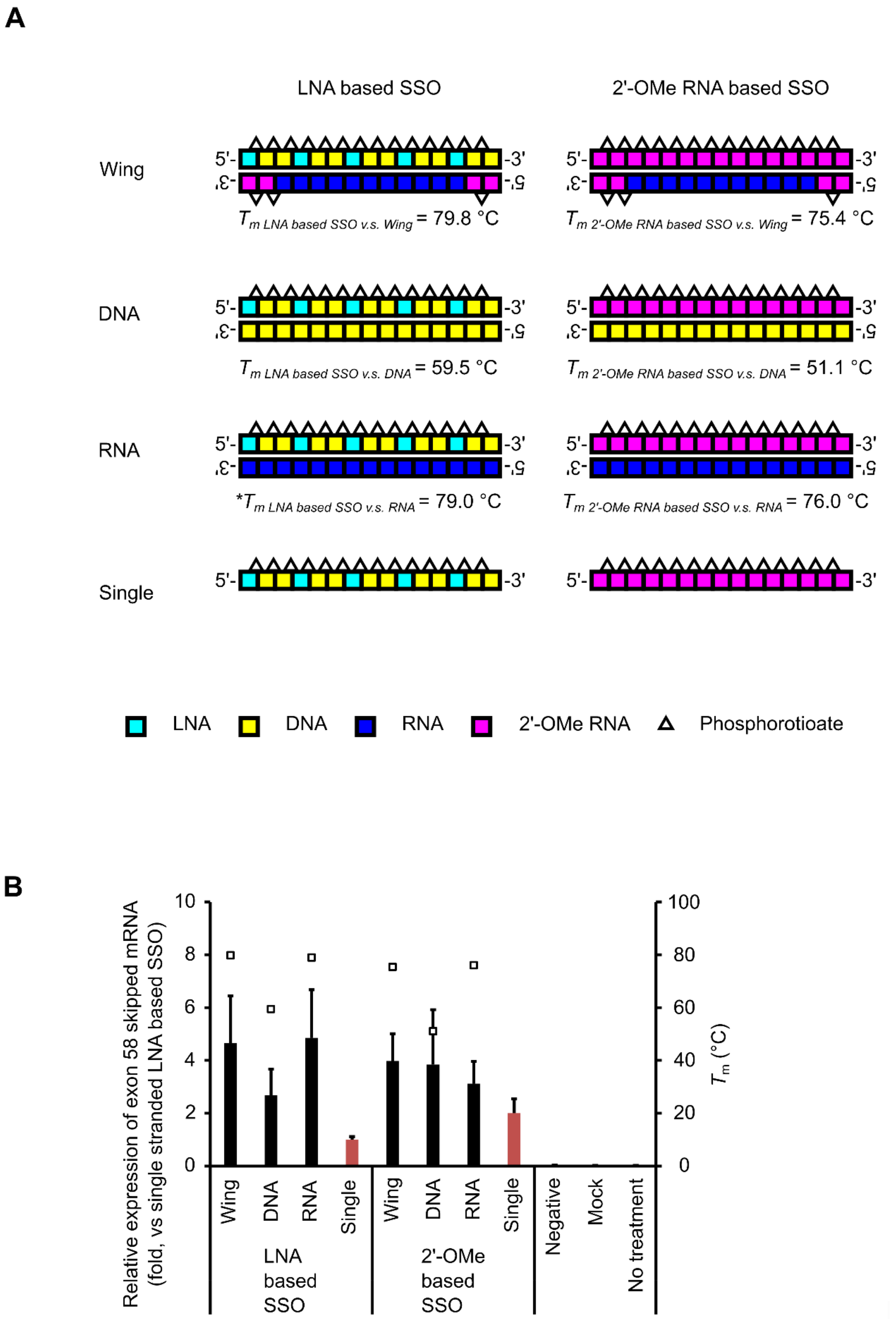
A
LNA based SSO
2'-OMe RNA based SSO
Wing
DNA
RNA
Single
5'-
-3'
$T_{m\ LNA\ based\ SSO\ v.s.\ Wing}$ = 79.8 °C
$T_{m\ 2'\text{-}OMe\ RNA\ based\ SSO\ v.s.\ Wing}$ = 75.4 °C
$T_{m\ LNA\ based\ SSO\ v.s.\ DNA}$ = 59.5 °C
$T_{m\ 2'\text{-}OMe\ RNA\ based\ SSO\ v.s.\ DNA}$ = 51.1 °C
*$T_{m\ LNA\ based\ SSO\ v.s.\ RNA}$ = 79.0 °C
$T_{m\ 2'\text{-}OMe\ RNA\ based\ SSO\ v.s.\ RNA}$ = 76.0 °C
LNA
DNA
RNA
2'-OMe RNA
Phosphorotioate
B
Relative expression of exon 58 skipped mRNA (fold, vs single stranded LNA based SSO)
T_m (°C)
0
2
4
6
8
10
0
20
40
60
80
100
Wing
DNA
RNA
Single
LNA based SSO
2'-OMe based SSO
Negative
Mock
No treatment

Figure S1 Evaluation of exon skipping activities of LNA based HDSSOs targeting 5'-site of *DMD* exon 58 in stable reporter cells.

(A) Schematic representation of the SSOs and complementary oligonucleotides used in the study. Each box shows that the one nucleotide; light blue: LNA, yellow: DNA, dark blue: RNA, red: 2'-OMe RNA. White triangles show phosphorothioate bonds. The melting temperatures of HDSSOs were also measured in at 4 μM HDSSO per 10 mM NaCl and 10 mM phosphate buffer (pH 7.2). Values represent the mean of three or four independent experiments performed. *Reference (1). (B) The result of exon skipping activities of each duplexed SSOs. The expression levels of *DMD* exon 58 skipped mRNA were measured by quantitative RT-PCR. The expression levels of *GAPDH* mRNA were used as an internal control. The graph shows the normalized *DMD* exon58 skipping activities, relative to the value in the single strand LNA based SSO-transfected cells (set at 1). Values represent the mean ± standard deviation of three independent experiments performed in duplicate. Negative; single-stranded LNA-based SSO that we reported previously as "LNA SSO (+10+24)",[1] was used as a negative control. Mock: treated with Lipofectamine RNAiMAX only; no treatment: no transfection. The white box shows the melting temperatures of HDSSOs.

FigureS2

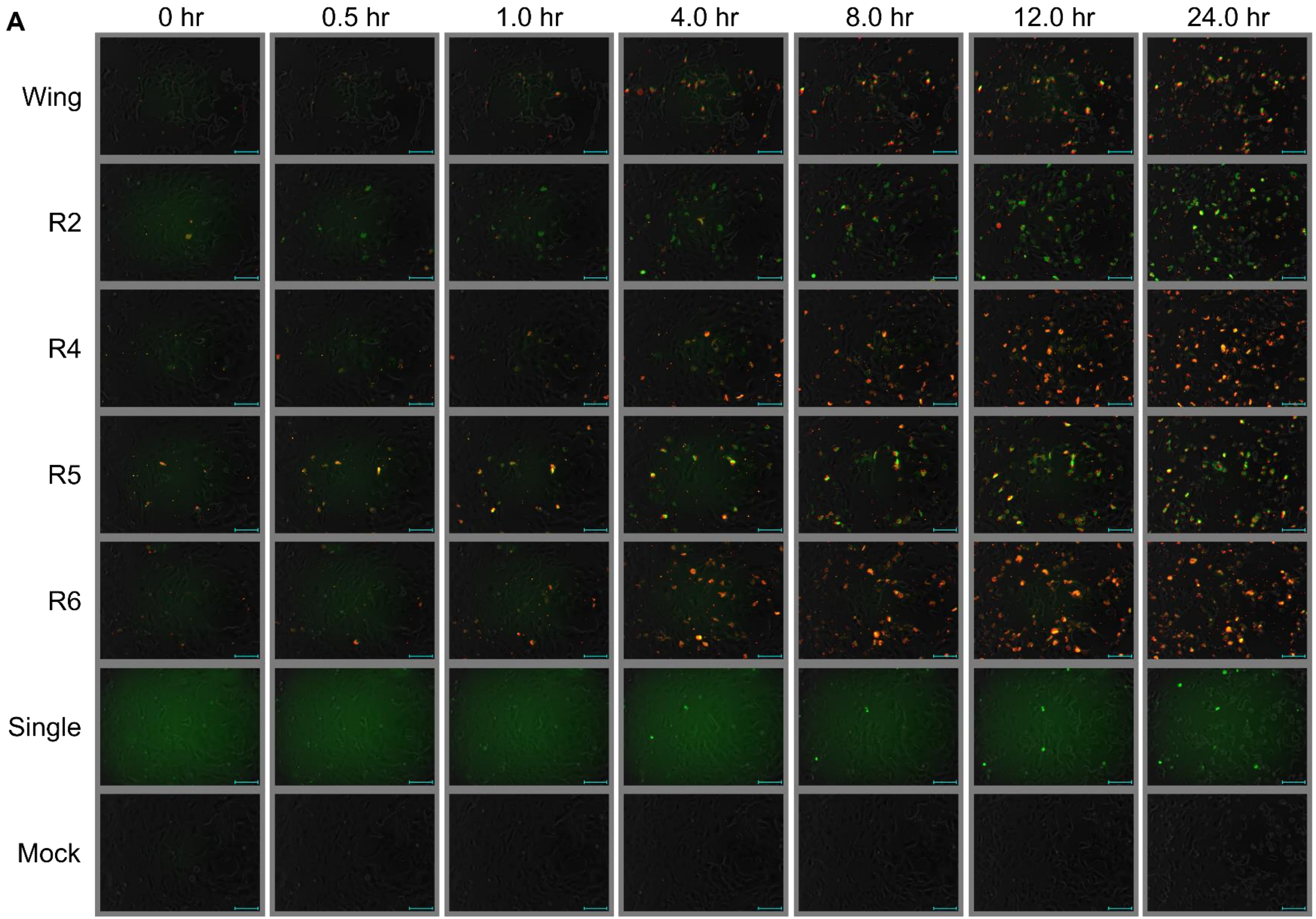
A
0 hr
0.5 hr
1.0 hr
4.0 hr
8.0 hr
12.0 hr
24.0 hr
Wing
R2
R4
R5
R6
Single
Mock

FigureS2 (continued)

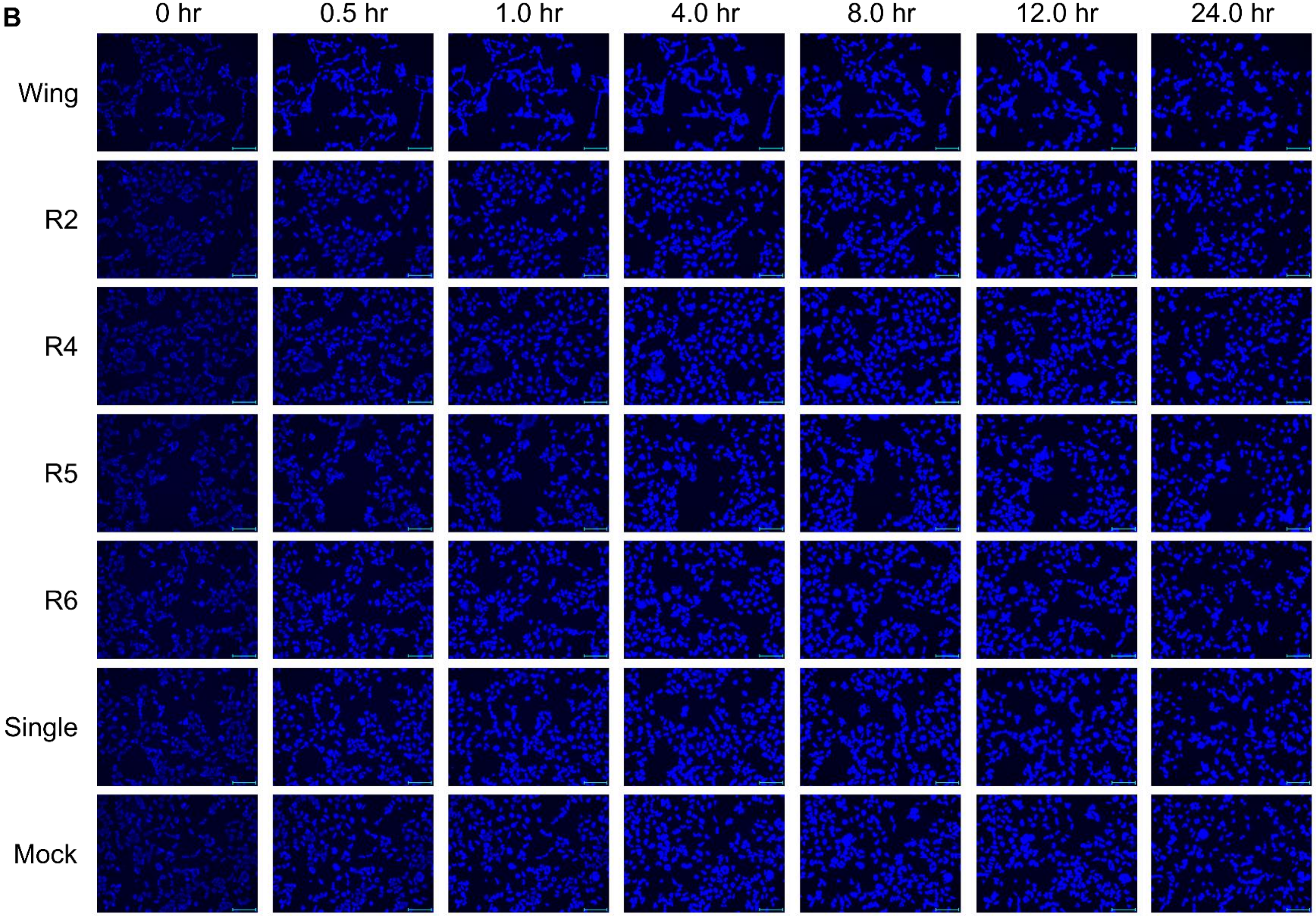

FigureS2 (continued)

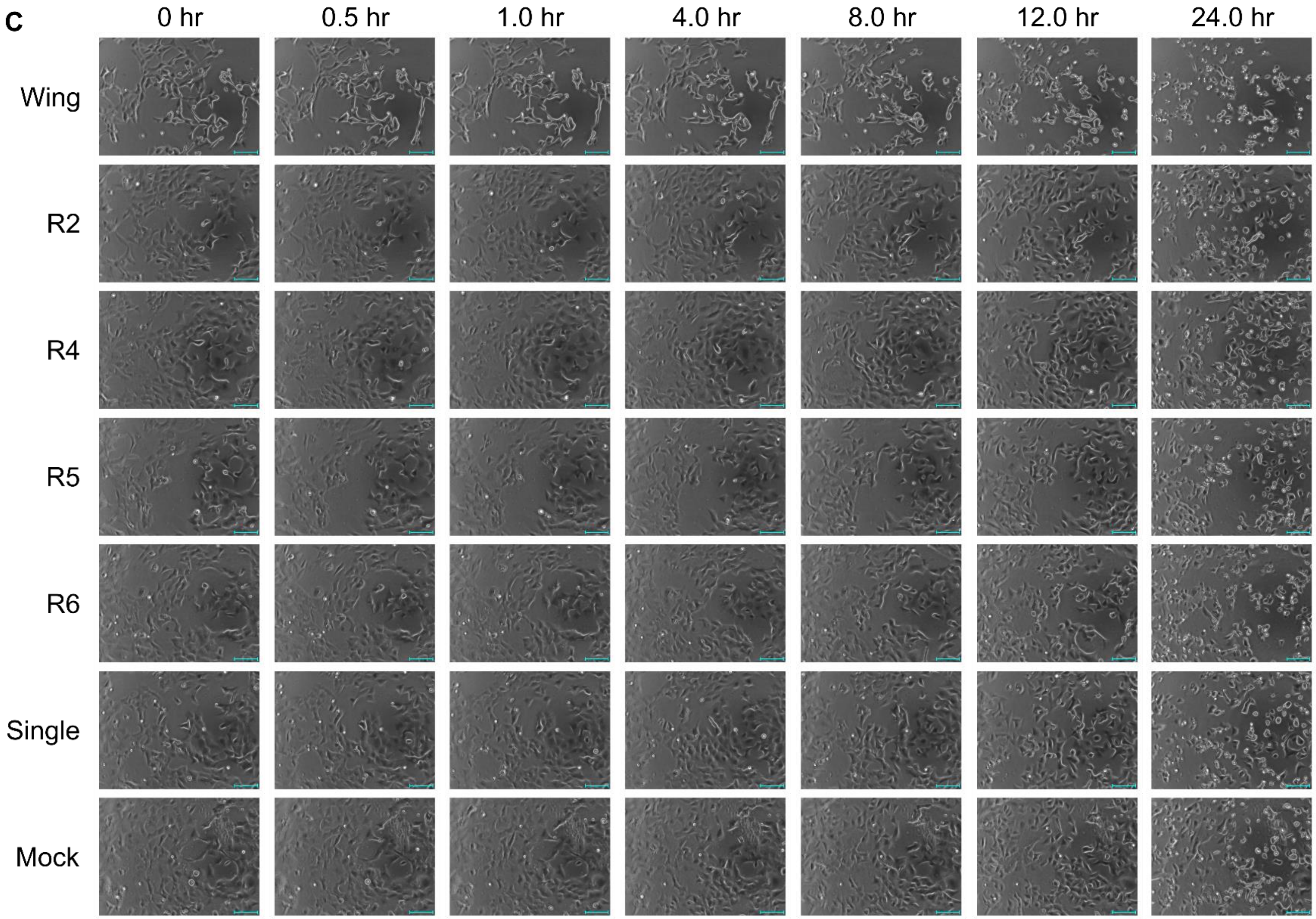

Figure S2 Time-lapse imaging of LNA based HDSSOs in HEK293 cells.

LNA based HDSSOs containing various complementary oligonucleotides were transfected into HEK293 cells using Lipofectamine RNAiMAX. Immediately after HDSSOs transfection, the images were captured with BZ-X700 (KEYENCE) at every 30 mins until 24 hr after transfection. (A-C) The captured images at 0, 0.5, 1.0, 4.0, 8.0, 12.0 and 24.0 h are shown. Green: 6-FAM conjugated SSOs, red: Alexa-647 conjugated complementary oligonucleotides, yellow: both 6-FAM conjugated SSOs and Alexa-647 conjugated complementary oligonucleotides were co-existed. Images in Figure 4 are part of this result. These images were obtained from an independent experiment performed on a different day than the experiment in both Figure 3 and Supplementary Video S1. Scale bars show 100 μm respectively. (A) The images, phase and both green and red fluorescence at same time-point, were merged using the BZ-X Analyzer software (KEYENCE). Images in Figure 4 are part of this result. (B) The images, blue fluorescence (Nuclei were stained using Hoechst 33342), are shown. (C) The images, phase contrast, are shown.

FigureS3

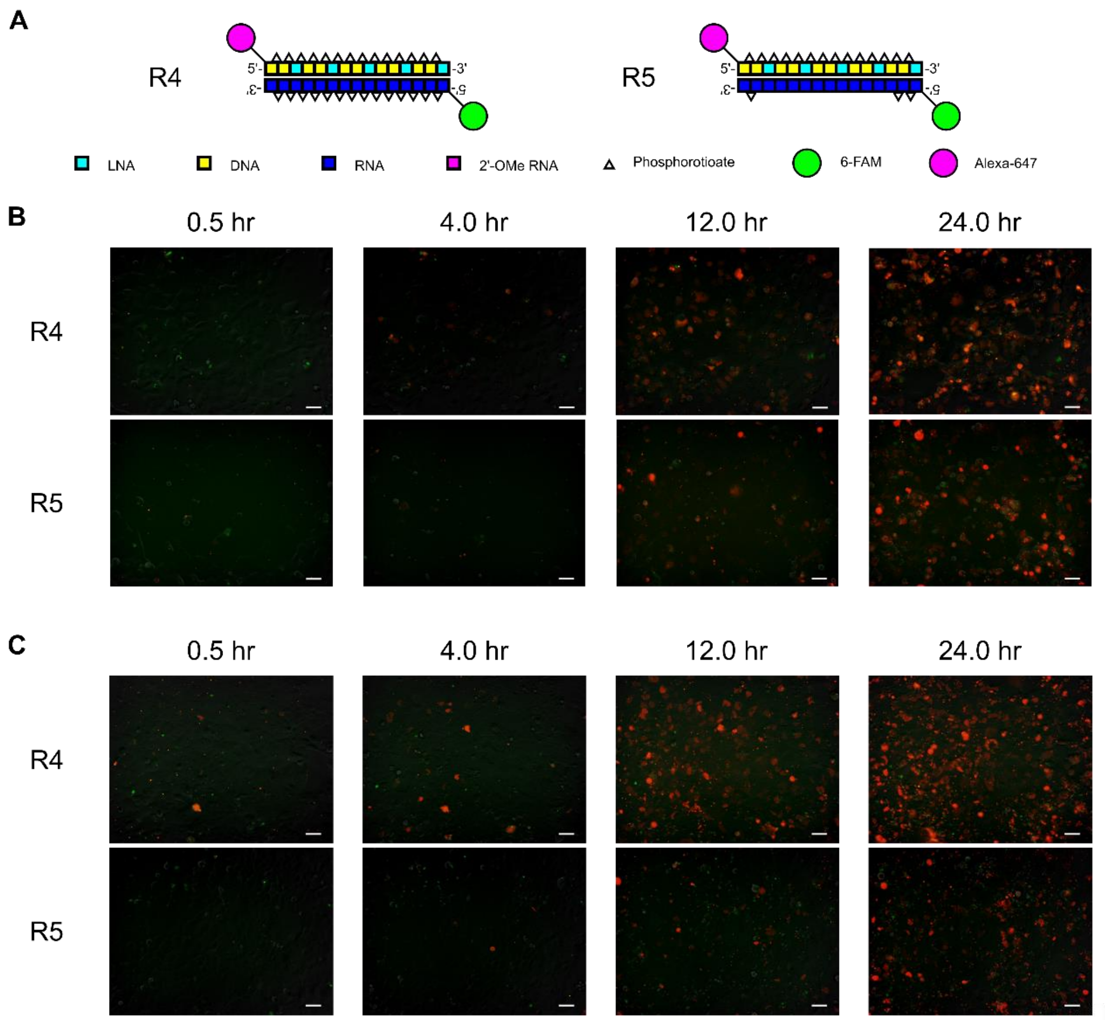

Figure S3 Time-lapse imaging of LNA-based HDSSOs in HEK293 cells with swapped fluorescent molecules.

(A) Schematic representation of the SSOs and complementary oligonucleotides used in the study. Each box shows one nucleotide; light blue: LNA, yellow: DNA, dark blue: RNA. White triangles show phosphorothioate bonds. Green: 6-FAM, red: Alexa-647. LNA-based HDSSOs containing the complementary oligonucleotides, R4 and R5, were transfected into HEK293 cells using Lipofectamine RNAiMAX. Immediately after transfection, images were captured with a BZ-X700 (KEYENCE) every 30 min until 24 h post-transfection. (B and C) The results for HDSSOs containing complementary oligonucleotides R4 and R5 are shown. Images captured at 0.5, 4.0, 12.0 and 24.0 h are shown. The images, phase, and both green and red fluorescence at the same time point were merged using BZ-X Analyzer software (KEYENCE). Red: Alexa-647 conjugated SSOs, green: 6-FAM conjugated complementary oligonucleotides; yellow: both Alexa-647 conjugated SSOs and 6-FAM conjugated complementary oligonucleotides co-existed. The

images in (B) and (C) were obtained from an independent experiment performed on a different day to show the reproducibility of results. Scale bars show 100 μm respectively.

FigureS4

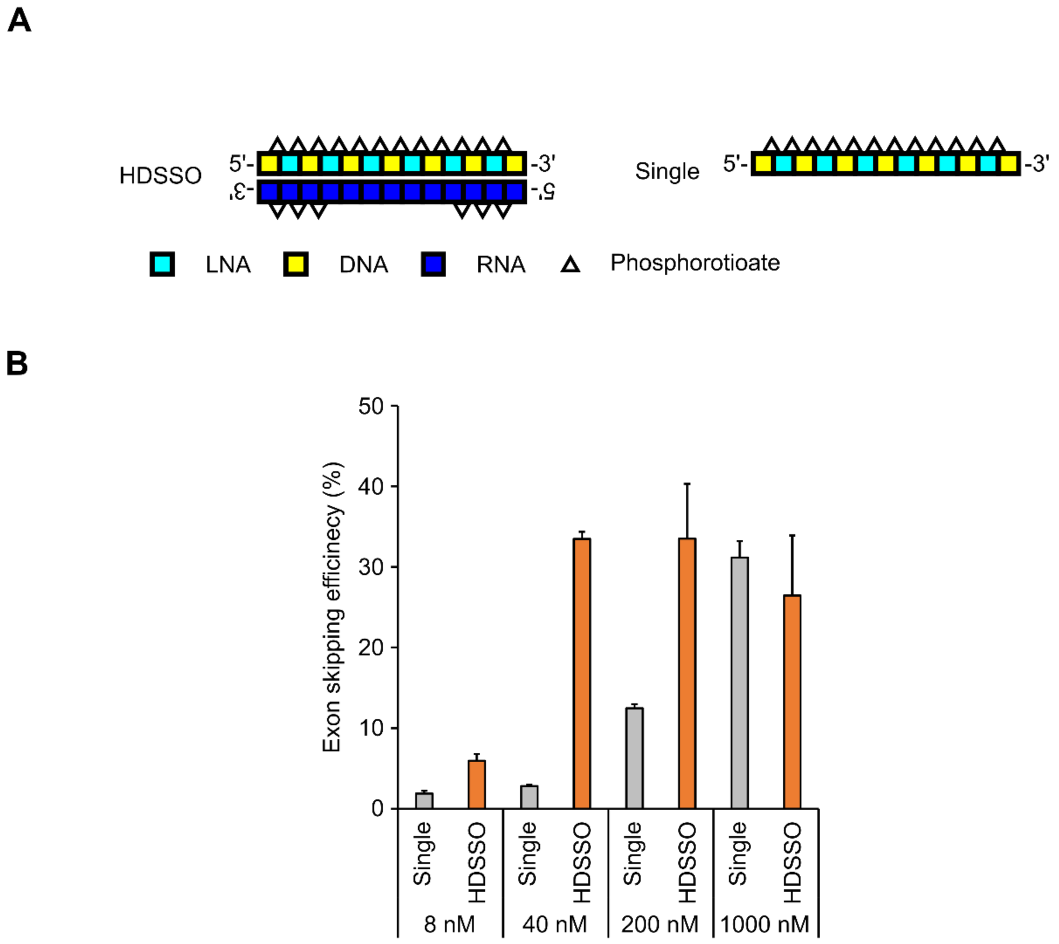

Figure S4 Evaluation of exon skipping activities of newly designed LNA based HDSSOs targeting 5'-site of *Dmd* exon 23 in C2C12 cells.

(A) Schematic representation of the SSOs and complementary oligonucleotides used in the study. Each box shows that the one nucleotide; light blue: LNA, yellow: DNA, dark blue: RNA. White triangles show phosphorothioate bonds. (B) The result of RT-PCR assay. In the 2% differentiation medium, C2C12 myotubes were incubated up to 48 hours. We then transfected Single or HDSSO (TableS7) against exon 23 of dystrophin gene at the final concentration of 8, 40, 200, or 1000 nM, respectively, with lipofectamine RNAiMAX (Thermo) and incubated them for 48 h. Then, cells were harvested. Total RNA was extracted from cells by ISOGEN 2 (NIPPON GENE) and 200 ng of total RNA was used for one-step RT-PCR (Qiagen) according to the manufacturer's instructions. The primer sequences were mEx22F 5'-ATCCAGCAGTCAGAAAGCAAA-3' and mEx24R 5'-CAGCCATCCATTTCTGTAAGG-3' for amplification from exons 22 to 24. The PCR conditions

were 50°C for 30 min and 95°C for 15 min, then 35 cycles of 94°C for 1 min, 60°C for 1 min, 72°C for 1 min, and finally 72°C for 7 min. The intensity of PCR bands was analyzed by using Bioanalayzer 2100 (Agilent), and skipping efficiency was calculated by using the following formula [(the intensity of skipped band)/(the intensity of skipped band + the intensity of unskipped band)]. Values represent the mean ± standard error of three independent experiments performed in duplicate.

FigureS5

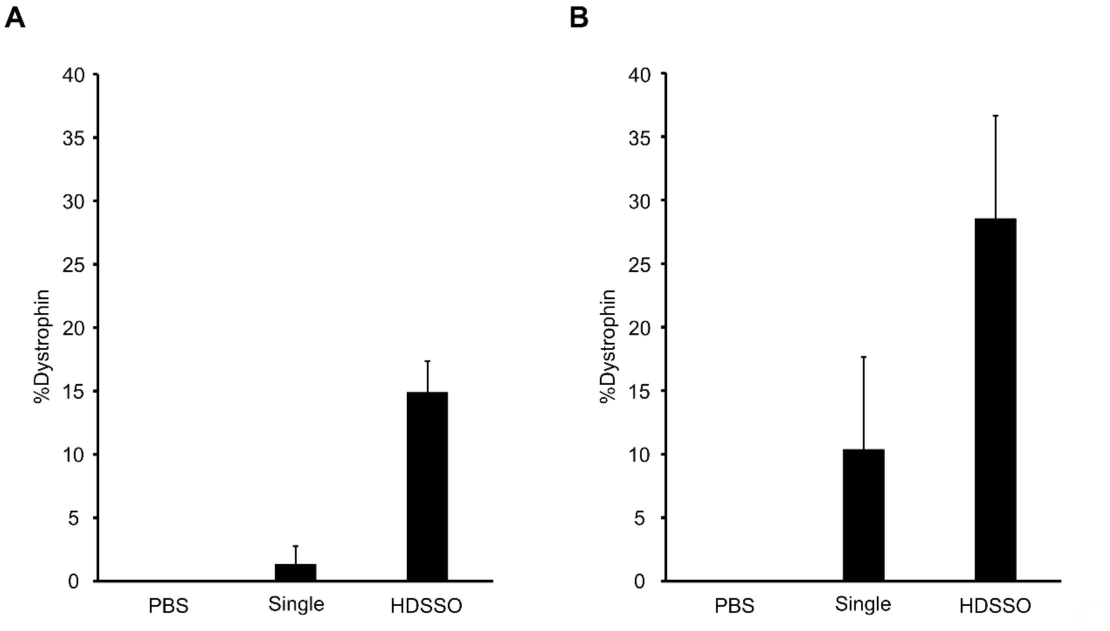

Figure S5 The exon skipping activity of LNA-based HDSSOs targeting the 5'-site of Dmd exon 23 in vivo using mdx mice.

Detection of dystrophin restoration by western blot analysis in the tibialis anterior muscle of mdx 2 weeks after intramuscular injection of SSO or HDSSO at A) 2 or B) 8 nmol compared to mdx and WT control B10 mice. The band intensities of dystrophin protein in Figure 5E of main text were detected as %dystrophin using ImageJ (fiji) software. The dystrophin expressions of WT control B10 mice was used for a calibration curve. Values represent the mean ± standard deviation (n=3 per each group).

FigureS6

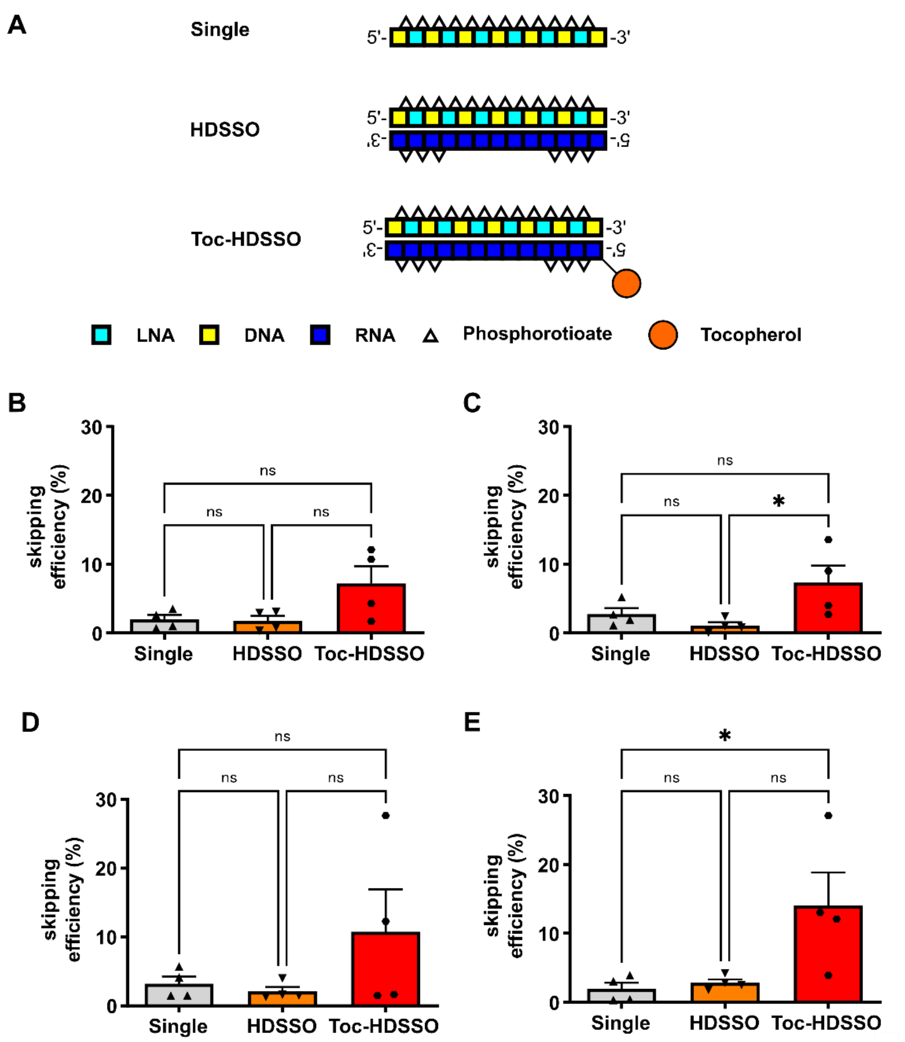

Figure S6 The exon skipping activity of LNA-based HDSSOs targeting the 5'-splice site of Dmd exon 23 in vivo using mdx mice under systemic subcutaneous injection.

(A) Schematic representation of the SSOs and complementary oligonucleotides used in the study. Each box shows one nucleotide; light blue: LNA, yellow: DNA, dark blue: RNA. White triangles show phosphorothioate bonds. (B-E) The exon skipping activity of LNA-based HDSSOs in mdx mice. Detection of exon 23–skipped dystrophin mRNA in the (B) heart, (C) diaphragm, (D) triceps and (E) quadriceps of mdx two weeks after subcutaneous injection of SSO or HDSSO at an equimolar dose 11.88 μmol/kg. Values represent the mean ± standard error (n=4 per each group). Significant differences compared to the Single were determined using Tukey's test. $*p < 0.05$.

Video S1 Merged time-lapse video of LNA-based HDSSOs in HEK293 cells.

LNA based HDSSOs containing various complementary oligonucleotides were transfected into HEK293 cells using Lipofectamine RNAiMAX. Immediately after HDSSOs transfection, the images were captured with BZ-X700 (KEYENCE) at every 30 mins until 24 hr after transfection. Green: 6-FAM conjugated SSOs, red: Alexa-647 conjugated complementary oligonucleotides, yellow: both 6-FAM conjugated SSOs and Alexa-647 conjugated complementary oligonucleotides were co-existed. The images, phase and both green and red fluorescence at same time-point, were merged using the BZ-X Analyzer software (KEYENCE). The time-lapse videos of merged images at every 30 mins are shown. Images in Figure 3 are part of this result. These images were obtained from an independent experiment performed on a different day than the experiment in both Figure 4 and Figures S2A-C. (A-E) Merged time-lapse video, HEK293 cells transfected with HDSSOs containing (A) Wing, (B) R2, (C) R4, (D) R5 and (E) R6 respectively, are shown. (F) Merged time-lapse video, HEK293 cells transfected with Single (single LNA modified SSOs), is shown. (G) Merged time-lapse video, HEK293 cells treated with Lipofectamine RNAiMAX only (mock), is shown. Scale bars show 100 μm respectively.

Reference

1. Shimo, T., Tachibana, K., Saito, K., Yoshida, T., Tomita, E., Waki, R., Yamamoto, T., Doi, T., Inoue, T., Kawakami, J. *et al.* (2014) Design and evaluation of locked nucleic acid-based splice-switching oligonucleotides in vitro. *Nucleic Acids Res*, **42**, 8174-8187.
