# Supplementary material for: Effect of chemical modification on the exon-skipping activity of heteroduplex oligonucleotides
Source: Mol Ther Nucleic Acids. 2025 Feb 1;36(1):102468. doi: 10.1016/j.omtn.2025.102468 (PMC11875208; doi:10.1016/j.omtn.2025.102468)
Supplement: Document S2. Article plus supplemental information [file mmc9.pdf]

Molecular Therapy
Nucleic Acids
Original Article

Effect of chemical modification on the exon-skipping activity of heteroduplex oligonucleotides

Takenori Shimo,[1,5] Juri Hasegawa,[2,5] Kotaro Yoshioka,[2] Yusuke Nakatsuji,[1] Kotomi Aso,[1] Keisuke Tachibana,[1] Tetsuya Nagata,[2] Takanori Yokota,[2] and Satoshi Obika[1,3,4]

[1]Graduate School of Pharmaceutical Sciences, Osaka University, 1-6 Yamadaoka, Suita-shi, Osaka 565-0871, Japan; [2]Department of Neurology and Neurological Science, Graduate School of Medical and Dental Sciences, Tokyo Medical and Dental University, 1-5-45 Yushima, Bunkyo-ku, Tokyo 113-8519, Japan; [3]National Institutes of Biomedical Innovation, Health and Nutrition (NIBIOHN), 7-6-8 Saito-Asagi, Ibaraki, Osaka 567-0085, Japan; [4]Institute for Open and Transdisciplinary Research Initiatives (OTRI), Osaka University, 1-1 Yamadaoka, Suita, Osaka 565-0871, Japan

We applied heteroduplex oligonucleotide (HDO) technology, which uses an oligonucleotide hybridized with a complementary strand, to efficiently deliver locked nucleic acid (LNA)-based splice-switching oligonucleotides (SSOs) to the nucleus. Using an *in vitro* assay involving cationic lipids, we revealed that HDO technology increased the exon-skipping activity of LNA-based SSOs. To assess the effect of heteroduplex SSOs (HDSSOs) on exon-skipping activity, we designed and evaluated various HDSSOs using a series of complementary oligonucleotides with different sugar chemistries (DNA, RNA, and LNA), linkages (phosphodiester; PO and phosphorothioate; PS linkages), and lengths. HDO with different complementary oligonucleotide designs demonstrated a variety of exon-skipping activities. Next, we investigated the intracellular behavior of HDOs, which seemed to affect their efficient exon-skipping activity. We found that HDO technology increased the uptake of both SSOs and complementary oligonucleotides into the nuclei. Additionally, a series of complementary oligonucleotides showed different intracellular stabilities, and complementary oligonucleotide design appears to be one of the key factors affecting efficient exon skipping. Finally, we examined the exon-skipping activity of HDSSOs in *mdx* mice and found that HDSSOs exhibited higher exon-skipping activity than single-stranded LNA-based SSOs in these mice under intramuscular injections.

INTRODUCTION

The modulation of pre-mRNA splicing is a promising therapeutic strategy for many genetic disorders, including Duchenne muscular dystrophy (DMD) and spinal muscular atrophy.[1–3] In addition, this strategy is able to be applied for individualized treatment, n-of-1 therapy, of a rare mutation or rare genetic diseases.[4–6] Splice-switching oligonucleotides (SSOs) enable the modulation of pre-mRNA splicing by binding to the target region of the pre-mRNA and blocking the binding of splicing factors. Since Dominski and Kole reported that SSOs can modulate splicing,[7] the design of SSO factors, such as length, guanosine-cytosine (GC) content, and target sites on pre-mRNAs, has been well studied.[8–12] In addition, SSO chemistry has been optimized by several researchers.[10,13–24] Recent studies have focused on the efficient delivery of SSOs, such as conjugation with peptides and small molecules.[25–33] One potential option for the efficient delivery of SSOs is the use of heteroduplex SSOs (HDSSOs). The effect of HDSSOs on the delivery rate of SSOs has been well studied both *in vitro* and *in vivo* since the 2000s. In 2001, Morcos first reported that HDSSOs with morpholino-based SSOs with complementary DNA (cDNA) strands enabled the introduction of SSOs into cells using cationic lipids.[34] Since morpholinos do not have phosphodiester linkers that donate a negative charge, the cDNA strand provides a negative charge for association with the cationic lipids. Since this report, many studies have applied this duplex of morpholinos and DNA in their experiments.[35,36] Astriab-Fisher et al. revealed that 2′-*O*-methyl (2′-OMe) RNA-based SSOs with cDNA strands were better internalized by the nuclei than naked 2′-OMe RNA SSOs *in vitro*; this was when Lipofectamine 2000 was used to transfect the SSOs.[37] However, to the best of our knowledge, HDSSOs have not been applied in locked nucleic acid (LNA)-based SSOs. Additionally, although HDSSO is a simple idea where main-strand oligonucleotides are annealed with complementary oligonucleotides, not enough is known about the design of complementary oligonucleotides. Therefore, we sought to optimize the complementary oligonucleotides in HDSSOs for efficient delivery into the nucleus, which is necessary for higher exon-skipping efficiency.

Received 6 March 2024; accepted 29 January 2025;
https://doi.org/10.1016/j.omtn.2025.102468.

[5]These authors contributed equally

Correspondence: Takanori Yokota, Department of Neurology and Neurological Science, Graduate School of Medical and Dental Sciences, Tokyo Medical and Dental University, 1-5-45 Yushima, Bunkyo-ku, Tokyo 113-8519, Japan.
E-mail: tak-yokota.nuro@tmd.ac.jp
Correspondence: Satoshi Obika, Graduate School of Pharmaceutical Sciences, Osaka University, 1-6 Yamadaoka, Suita-shi, Osaka 565-0871, Japan.
E-mail: obika@phs.osaka-u.ac.jp

Published by Elsevier Inc. on behalf of The American Society of Gene and Cell Therapy.
This is an open access article under the CC BY license (http://creativecommons.org/licenses/by/4.0/).

Another aspect of HDSSOs is that it is possible to conjugate delivery reagents with complementary oligonucleotides. In 2015, Nishina et al. revealed that heteroduplex oligonucleotides can be applied in LNA-based gapmers (heteroduplex antisense oligonucleotides; HDASOs).[38] According to a previous report, complementary oligonucleotides in HDASO can be conjugated with α-tocopherol (Toc). As conjugation with various functional ligands suppresses antisense activities by inhibiting hybridization with target DNA,[39] complementary oligonucleotides in HDASOs seem to be promising carriers for ligands.

In this study, we showed that the design of complementary oligonucleotides for LNA-based SSOs in HDSSOs greatly affected their exon-skipping activity *in vitro*. According to our experiments, the design of complementary oligonucleotides affected exon-skipping activity. We also investigated the intracellular behavior of HDSSOs in HEK293 cells using time-lapse microscopy. These experiments showed that the intracellular stability of complementary oligonucleotides likely accounts for the differences in exon-skipping activity between the various complementary oligonucleotide designs. We also evaluated the effectiveness of HDSSOs *in vivo*. We found that HDSSOs had a higher exon-skipping activity than LNA-based SSO without complementary oligonucleotides under intramuscular injections.

RESULTS

Evaluation of LNA-based HDSSOs

Although HDSSOs consisting of either phosphorodiamidate morpholino oligomer (PMO)-based SSOs and cDNA strands or 2′-OMe RNA-based SSOs and cDNA strands have been studied,[34–37] there are no reports on HDSSOs consisting of LNA-based SSOs and complementary strands. To investigate whether HDSSOs could also be applied in LNA-based SSOs, we designed three complementary oligonucleotides for each SSO (Figure 1A; Table S1) with different chemistries (Wing, DNA, and RNA). Although previous studies with HDSSOs only used either native DNA-based or 2′-OMe RNA-based oligonucleotides as complementary oligonucleotides,[34–37] we thought that DNA, RNA, and other chemically modified strands might be functional because Nagata et al. and other groups previously reported that cRNA partially substituted with 2′-OMe RNA phosphorothioate (PS) (here called Wing) increased the activities of LNA-based gapmer ASOs, although they did not investigate HDSSOs.[38,40,41] Therefore, we used both Wing and naked RNA-based complementary oligonucleotides.

We evaluated the *in vitro* exon-skipping activity of LNA- and 2′-OMe RNA-based SSOs targeting the *DMD* gene (Figure 1A; Table S2) using stable reporter cells.[10] Quantitative RT-PCR (RT-qPCR) analysis revealed that LNA-based HDSSOs showed a higher exon-skipping efficiency than single-stranded LNA-based SSOs when using the cationic lipid Lipofectamine RNAiMAX (Figure 1B). Specifically, the exon-skipping activity of LNA-based HDSSOs with complementary Wing or RNA oligonucleotides was higher than that of LNA-based HDSSOs with cDNA oligonucleotides. In contrast, the exon-skipping activities of 2′-OMe RNA-based HDSSOs with cDNA oligonucleotides were similar to that of 2′-OMe RNA-based HDSSOs with Wing or RNA. We also determined the melting temperature (T_m) of the SSOs with complementary oligonucleotides. Ultraviolet (UV) melting experiments showed that the T_m value of DNA was lower than that of Wing or RNA. To confirm the generality, we also conducted *in vitro* assays with both LNA- and 2′-OMe RNA-based SSOs targeting other sites (5′-splice site) of the *DMD* gene and revealed the same tendency (Figure S1; Tables S3 and S4).

Structure-activity relationships of complementary oligonucleotides in HDSSOs

The results presented in Figures 1 and S1 imply that the HDSSOs technology could be applied in LNA-based SSOs. To date, the structure-activity relationship of complementary oligonucleotides in HDSSOs has not been well studied. Therefore, we synthesized various complementary oligonucleotides, including length, PS linkages, and base modifications to investigate the factors associated with the design of complementary oligonucleotides (Figure 2A; Table S5).

We designed group 1 complementary oligonucleotides, D1–3 and R1–3, of different lengths (9-, 15-, and 21-mer) and evaluated how the exon-skipping activities of the LNA-based SSOs varied with length. Among the DNA-based complementary oligonucleotides, there were no statistically significant differences between the HDSSOs with DNA-based complementary oligonucleotides D1 (21-mer) and D2 (15-mer); however, there were differences between the HDSSOs with D3 (9-mer) complementary oligonucleotides ($p < 0.05$ D1 versus D3, $p < 0.01$ D2 versus D3). An HDSSO with D3 (9-mer) had at least 1.35 times more exon-skipping activity than that of HDSSOs with 15- and 21-mer cDNA strands (D2 and D1, respectively) (Figure 2B). However, there were no statistically significant differences between the HDSSOs with RNA-based complementary oligonucleotides R1 (21-mer), R2 (15-mer), and R3 (9-mer).

Next, group 2 complementary oligonucleotides, D4–6 and R4–6, were designed to evaluate the effects of PS linkages and the introduction of LNA modification to increase nuclease resistance. D4 and R4 are fully PS linked, whereas D5–6 and R5–6 have a PS linkage at their 5′ and 3′ ends (D6 and R6 also have LNA modifications). Surprisingly, the exon-skipping activities of HDSSOs with D4–6 and R4 were notably lower than those of other designs, having almost the same or lower exon-skipping activities as single-stranded SSOs. In contrast, HDSSO with R5 showed the same exon-skipping activity as HDSSOs with complementary oligonucleotides without PS linkages (R2). HDSSO with R6 had lower exon-skipping activity than HDSSO with R5 ($p < 0.05$ R5 versus R6); however, it was still about five times higher than that of single-stranded SSOs ($p < 0.01$ Single versus R6).

Finally, to modulate the duplex stability of HDSSOs, we designed group 3 complementary oligonucleotides D7 and R7. We estimated that the low binding affinity of HDSSOs might result in higher exon-skipping activities because SSOs need to dissociate with complementary oligonucleotides to bind target mRNA after delivery to the nucleus. The complementary oligonucleotides D7 and R7 have a guanosine-to-inosine (G > I) substitution, which is expected to decrease

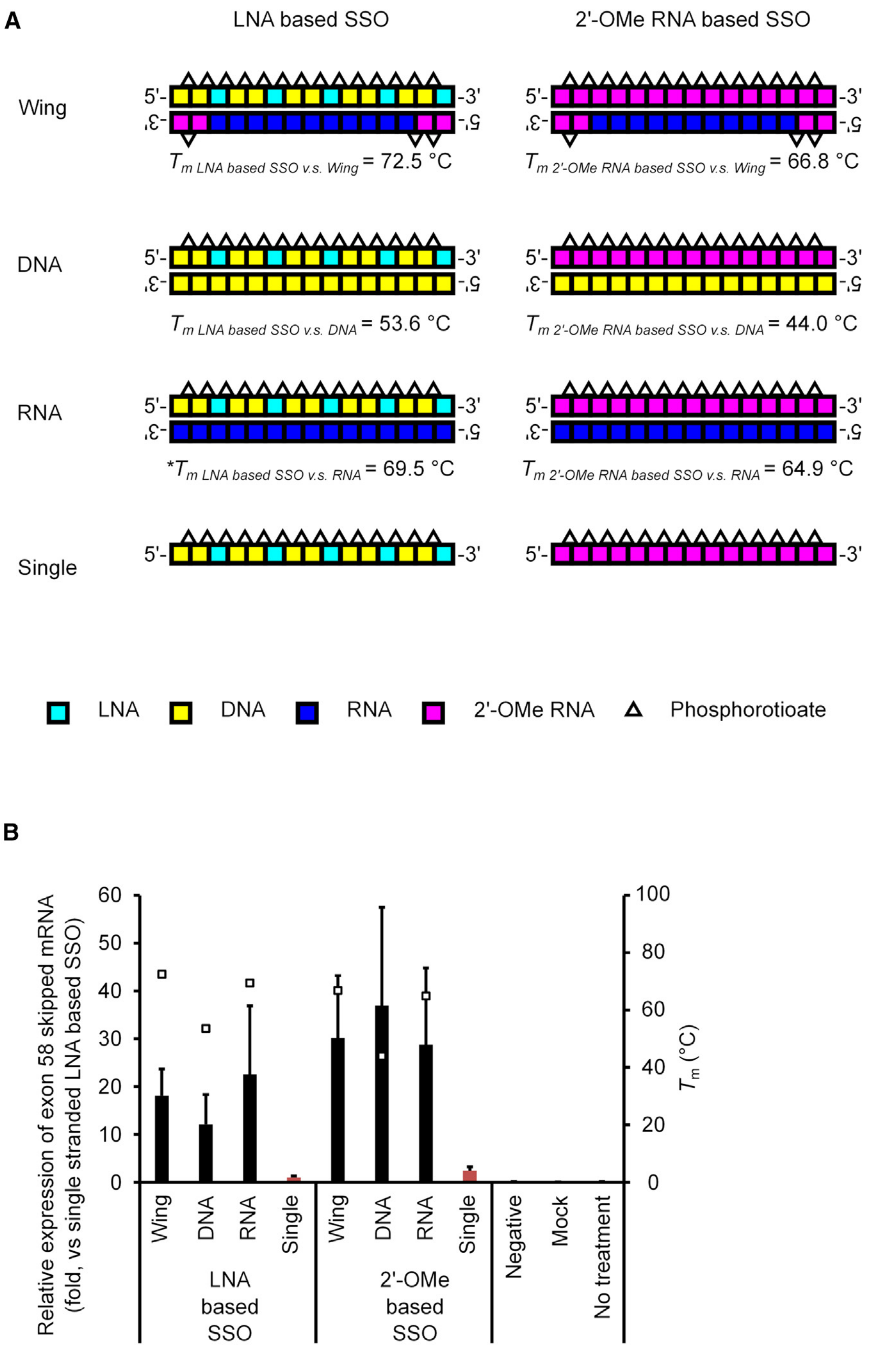

Figure 1. Evaluation of the exon-skipping activity of LNA-based HDSSOs targeting the 3′ site of the *DMD* exon 58 *in vitro* using stable reporter cells

(A) The SSOs and complementary oligonucleotides used in the study. Each box shows one nucleotide; light blue: LNA, yellow: DNA, dark blue: RNA, red: 2′-OMe RNA. White triangles show phosphorothioate bonds. The melting temperatures (T_m) of HDSSOs were also measured at 4 μM HDSSO per 10 mM NaCl and 10 mM phosphate buffer (pH 7.2). Values represent the mean of three or four independent experiments performed. (B) The exon-skipping activity of each HDSSO. The expression levels of *DMD* exon 58-skipped mRNA were measured by quantitative RT-PCR. The expression levels of *GAPDH* mRNA were used as an internal control. The graph shows the normalized *DMD* exon 58-skipping activities, relative to the value in the single-stranded LNA based SSO-transfected cells (set at 1). Values represent the mean ± standard deviation of three independent experiments performed in duplicate. Negative: single-stranded LNA-based SSO that we reported previously as "LNA SSO (+10 + 24)"[10] was used as a negative control. Mock: treated with Lipofectamine RNAiMAX only; no treatment: no transfection. The white box shows the T_m of HDSSOs.

the T_m of the inosine-cytosine base pair compared to the GC base pair.[20,42] Therefore, all guanosines were replaced with inosines in the complementary oligonucleotides. Using these complementary oligonucleotides, we evaluated the exon-skipping activity of HDSSOs. HDSSOs with the complementary oligonucleotide D7 showed higher exon-skipping activity than that of HDSSOs with the complementary oligonucleotide D2 ($p < 0.001$ D2 versus D7, approximately 10 times higher than single-stranded SSO, $p < 0.001$ Single versus D7). The HDSSOs with RNA-based complementary oligonucleotides, R7, also showed high exon-skipping activity (approximately nine times higher than that of single-stranded SSOs) ($p < 0.01$ Single versus R7); however, the exon-skipping activity of HDSSOs with the other RNA-based complementary oligonucleotides such as R2 was the same as that of HDSSOs with R7. Thus, the G > I substitutions enabled us to increase the exon-skipping activity of HDSSOs with the DNA-based complementary oligonucleotide ($p < 0.001$ D2 versus D7), but it did not increase the exon-skipping activity of the HDSSOs with the RNA-based complementary oligonucleotide.

Visualizing the intracellular behavior of LNA-based HDSSOs using time-lapse microscopy imaging

We confirmed that LNA-based HDSSOs exhibited increased exon-skipping activity, as shown in Figures 1 and 2. Additionally, we found that different designs of complementary oligonucleotides affected the exon-skipping activities of SSOs (Figure 2). We speculated that the intracellular localization of SSOs was affected by the design of complementary oligonucleotides in HDSSOs according to a previous report.[43] To understand this better, we investigated the intracellular behavior of HDSSOs with group 2 complementary oligonucleotides (evaluating the effects of PS linkages and introducing LNA modification to

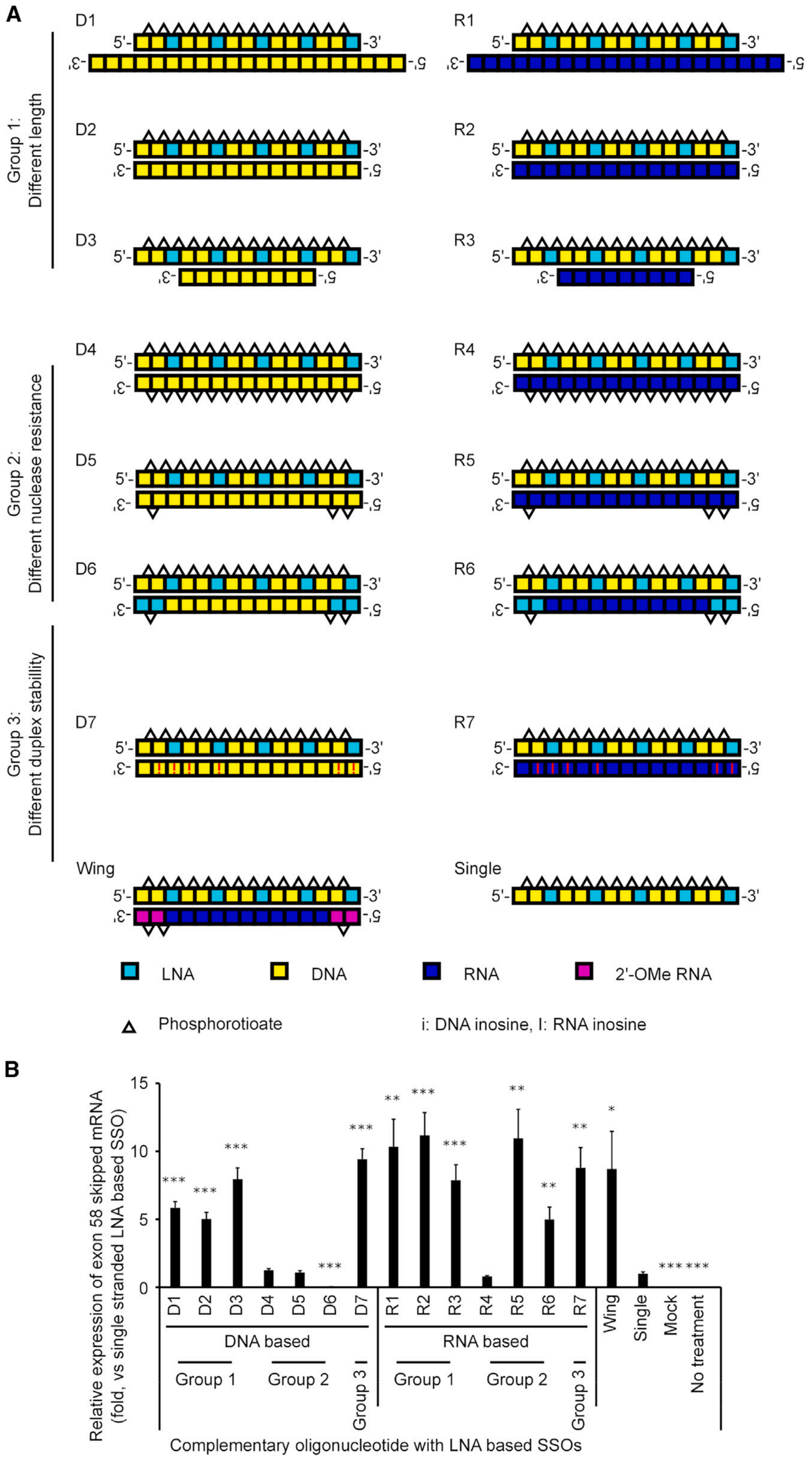

Figure 2. Screening of complementary oligonucleotide design and evaluation of the exon-skipping activity of LNA-based HDSSOs

(A) Schematic representation of the SSOs and complementary oligonucleotides used in the study. D2 and R2 are also called "DNA" and "RNA" in Figure 1, respectively. Each box shows one nucleotide; light blue: LNA, yellow: DNA, dark blue: RNA, red: 2′-OMe RNA. White triangles show phosphorothioate bonds. i, DNA inosine; I, RNA inosine. (B) The exon-skipping activity of each HDSSO. The expression level of *DMD* exon 58-skipped mRNA was measured by quantitative RT-PCR. The expression level of *RPLP2* mRNA was used as an internal control. The graph shows the normalized *DMD* exon 58-skipping activities, relative to the value in the single-stranded SSO-transfected cells (set at 1). The red graph shows the result of single-stranded SSO-transfected cells. The blue graphs show the results of HDSSOs also used for the assay in Figure 1; D2, DNA; R2, RNA; and Wing, Wing in Figure 1, respectively. Values represent the mean ± standard deviation of three independent experiments performed in duplicate. Significant differences were analyzed by one-way ANOVA followed by Dunnett's T3 test, and those compared with the single-stranded SSO group are indicated by asterisks (*$p < 0.05$; **$p < 0.01$; ***$p < 0.001$). Mock, treated with Lipofectamine RNAiMAX only; no treatment: no transfection.

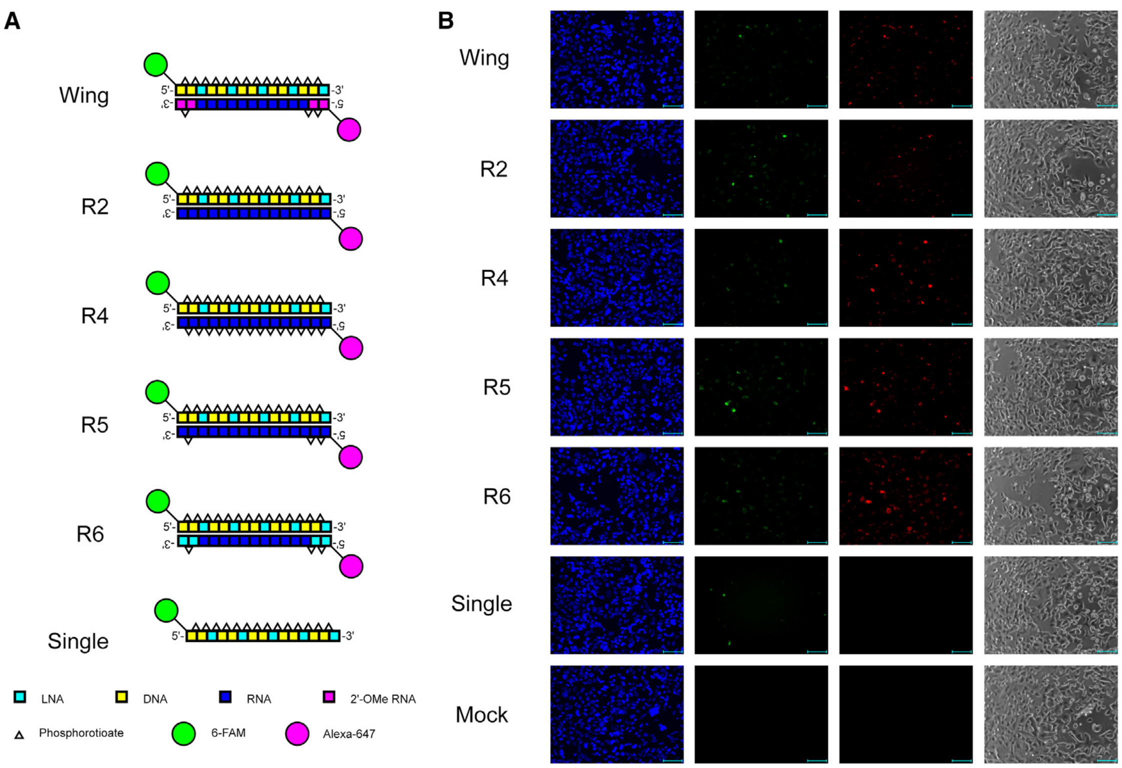

Figure 3. Time-lapse imaging of LNA-based HDSSOs in HEK293 cells

LNA-based HDSSOs containing various complementary oligonucleotides were transfected into HEK293 cells using Lipofectamine RNAiMAX. Immediately after transfection, images were captured with a BZ-X700 (Keyence) every 30 min until 24 h post-transfection. (A) Schematic representation of the SSOs and complementary oligonucleotides used in the study. Each box shows one nucleotide; light blue: LNA, yellow: DNA, dark blue: RNA, red: 2′-OMe RNA. White triangles show phosphorothioate bonds. Green: 6-FAM, red: Alexa 647. (B) The captured images at 24 h are shown. The images, green fluorescence, red fluorescence, blue fluorescence (nuclei were stained using Hoechst 33342), and phase contrast are shown. Green, 6-FAM conjugated SSOs; red, Alexa 647 conjugated complementary oligonucleotides. These images were obtained from an independent experiment performed on a different day than the experiment in Figure 4. More detailed and merged data are presented in Videos S1A, S1B, S1C, S1D, S1E, S1F, and S1G. Scale bars, 100 μm.

increase nuclease resistance) R4–6 using time-lapse microscopy imaging (Figures 3, 4, and S2 and Videos S1A, S1B, S1C, S1D, S1E, S1F, and S1G). As controls, we also investigated HDSSOs with R2, Wing, and single-stranded LNA-based SSOs (Single). Both LNA-based SSOs and complementary oligonucleotides were labeled with the fluorescent molecules 6-FAM (green fluorescence) for LNA-based SSOs and Alexa 647 (red fluorescence) for complementary oligonucleotides (Figure 3A; Table S6). After transfection of HDSSOs or single-stranded SSOs using cationic lipid Lipofectamine RNAiMAX, fluorescent images were captured every 30 min for 24 h post-transfection. Both green and red fluorescence were detected in the nuclei of many cells transfected with all types of HDSSOs after 30 min (Figure S2A; Videos S1A, S1B, S1C, S1D, S1E, S1F, and S1G). This showed that both SSOs and complementary oligonucleotides were immediately localized to the nuclei, although it remains unknown whether SSOs and complementary oligonucleotides formed a duplex. At 24 h after transfection, cells expressing green fluorescence were more frequently detected in the nuclei of cells transfected with HDSSOs rather than with single-stranded LNA-based SSOs (Single) (Figure 3B). In particular, HDSSOs containing R2 and R5, which showed higher exon-skipping activities than single-stranded LNA-based SSOs (Figure 2), induced a higher number of cells expressing green fluorescence.

From 0.5 to 24 h after transfection, the red fluorescence in the nuclei of cells transfected with HDSSOs containing R2, R5, and Wing began to disappear (Figures 4 and S2A; Videos S1A, S1B, S1C, S1D, S1E, S1F, and S1G). In fact, 24 h after transfection, less red fluorescence was detected in the nuclei of cells transfected with HDSSOs containing R2, R5, and Wing than other complementary oligonucleotides (Figures 3B, 4, and S2A; Videos S1A, S1B, S1C, S1D, S1E, S1F, and S1G). In addition, cells transfected with HDSSOs containing R2, R5, and Wing showed granules of red fluorescence (Figures 3B, 4, and S2A; Videos S1A, S1B, S1C, S1D, S1E, S1F, and S1G). This indicated that the complementary oligonucleotides R2, R5, and Wing were digested or excluded from the nuclei. However, the green fluorescence in the nuclei did not disappear, even though the HDSSOs had different complementary oligonucleotide designs (Figures 3B, 4, and S2A; Videos S1A, S1B, S1C, S1D, S1E, S1F, and S1G). Therefore, we theorized that only the complementary oligonucleotides

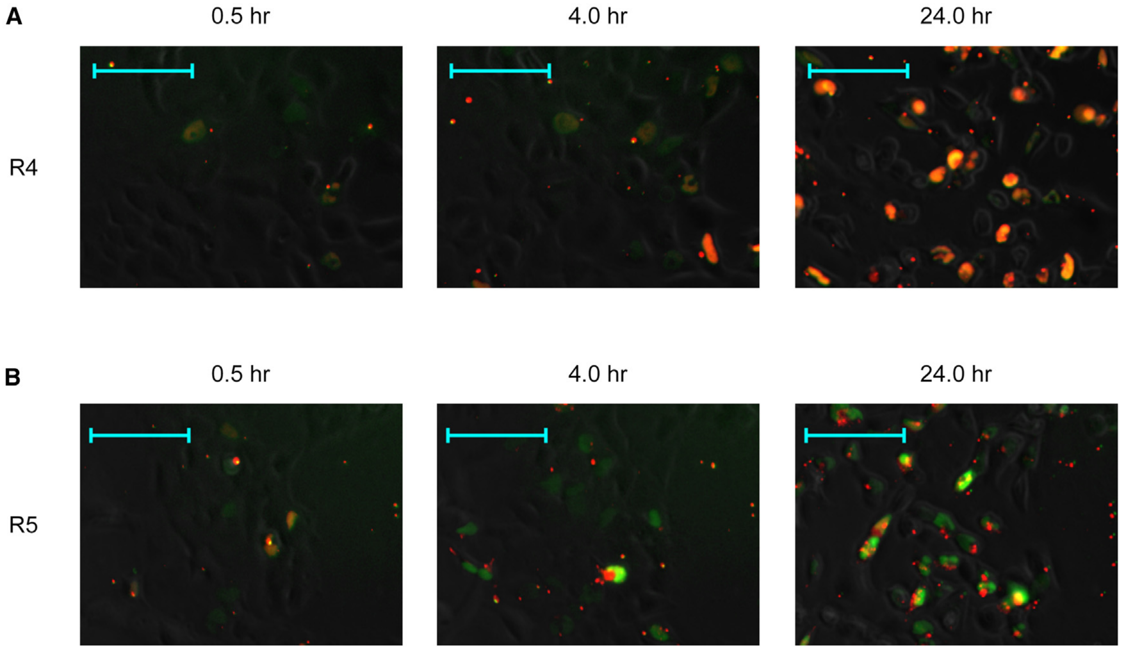

Figure 4. Merged time-lapse imaging of LNA-based HDSSOs containing R4 and R5 in HEK293 cells
LNA-based HDSSOs containing the complementary oligonucleotides R4 and R5 were transfected into HEK293 cells using Lipofectamine RNAiMAX. Immediately after HDSSOs transfection, the images were captured with BZ-X700 (Keyence) every 30 min for 24 h after transfection. The results for HDSSOs containing complementary oligonucleotides (A) R4 and (B) R5 are shown. (A and B) Images captured at 0.5, 4.0, and 24.0 h are shown. The images, phase, and both green and red fluorescence at the same time point were merged using BZ-X Analyzer software (Keyence). Green: 6-FAM conjugated SSOs; red: Alexa 647 conjugated complementary oligonucleotides; yellow: both 6-FAM conjugated SSOs and Alexa 647 conjugated complementary oligonucleotides co-existed. These images were obtained from an independent experiment performed on a different day than the experiment in Figure 3. Images in Figure 4 are part of the result from Figure S2A. More detailed data are presented in Figure S2. Scale bars, 100 μm.

were either digested in the nuclei or excluded from the nuclei, whereas the SSOs remained in the nuclei. Additionally, the disappearance rate of complementary oligonucleotides seemed to be one of the factors that differed in the exon-skipping activities of HDSSOs, although it is still unknown whether LNA-based SSOs bind to complementary oligonucleotides that exist in the nuclei. In fact, many cells transfected with HDSSOs containing R4 and R6 retained red fluorescence in the nuclei even at 24 h after transfection, and these cells showed low exon-skipping activities (Figures 2B, 3B, 4, and S2A; Videos S1A, S1B, S1C, S1D, S1E, S1F, and S1G). To replicate the experiment investigating the intracellular behavior of HDSSOs, we also swapped the fluorescent molecules of HDSSOs containing R4 and R5 (Figure S3). Thus, both LNA-based SSOs and complementary oligonucleotides were labeled with the fluorescent molecules Alexa 647 (red fluorescence) for LNA-based SSOs and 6-FAM (green fluorescence) for complementary oligonucleotides. The result showed the same tendency; many cells transfected with HDSSOs containing R4 retained green fluorescence in the nuclei even at 24 h after transfection, but the cells transfected with HDSSOs containing R5 did not.

Evaluation of the *in vivo* activity of HDSSOs

We showed in Figures 1 and 2 that LNA-based HDSSOs had increased exon-skipping activity *in vitro*. In addition, the different designs of complementary oligonucleotides affect the intracellular behavior of LNA-based SSOs, as shown in Figures 3, 4, and S2 and Videos S1A, S1B, S1C, S1D, S1E, S1F, and S1G. All things considered, we showed that the design of the complementary oligonucleotides affected their exon-skipping activity by modifying the intracellular behavior and that the RNA-based complementary oligonucleotide R5, which had a partial PS backbone at both the 5′ and 3′ ends, efficiently enhanced the exon-skipping activity of SSOs *in vitro*. Therefore, we investigated the exon-skipping activity of HDSSOs using complementary oligonucleotides based on R5 design *in vivo*. We used *mdx* mice for the *in vivo* assays; therefore, the sequence of LNA-based SSOs was redesigned for the mouse *Dmd* exon 23 skipping (Table S7). According to previous reports,[44–46] the designed LNA-based SSOs target the site of the mouse *Dmd* exon 23-intron 23 junction. Also, the designed LNA-based SSO has a length of 13-mer with six LNA analogs according to our previous report.[10] Before conducting the *in vivo* assay, we investigated the exon-skipping activity of LNA-based SSOs against the mouse *Dmd* gene *in vitro* using C2C12 cells (Figure S4). The newly designed HDSSOs, with a cRNA strand and a partial PS backbone at both the 5′ and 3′ ends, had higher exon-skipping activities than single-stranded SSOs at 8, 40, and 200 nM.

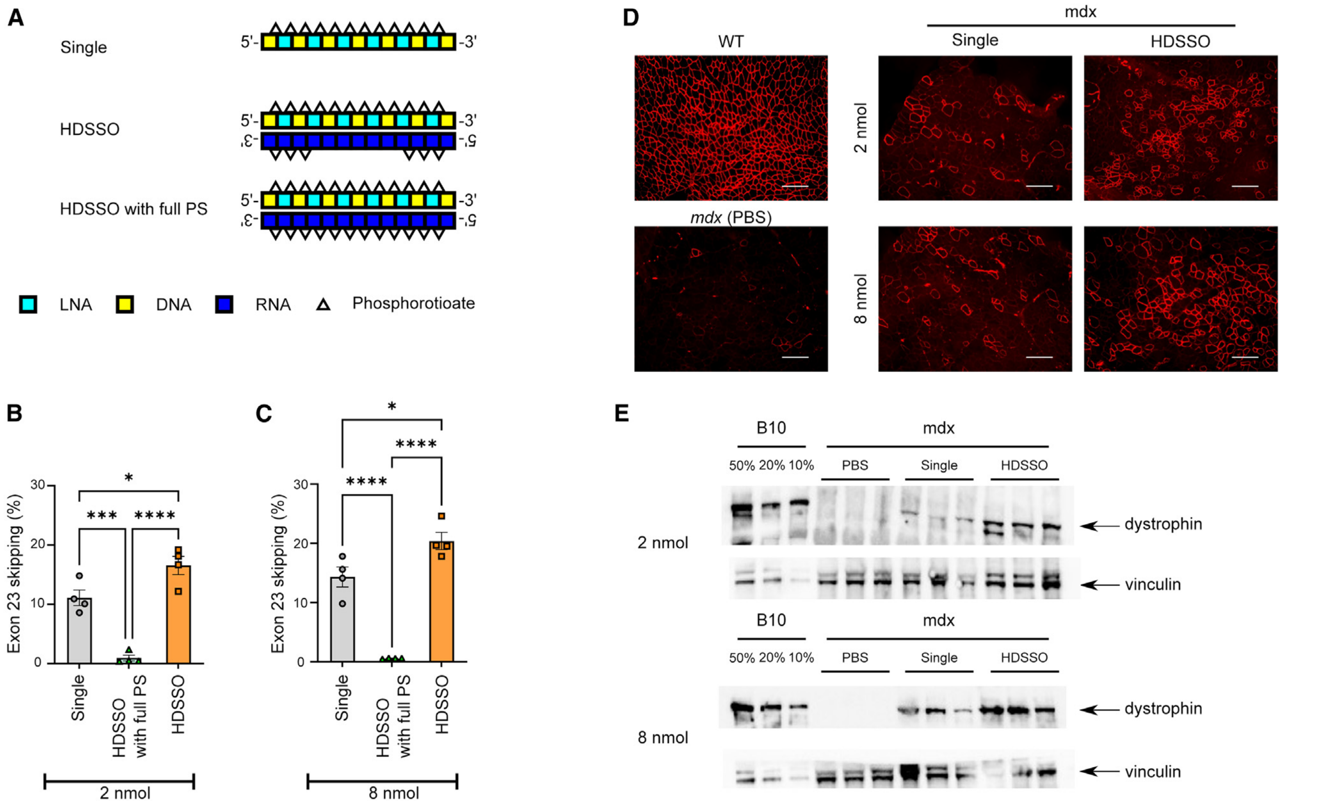

Figure 5. The exon-skipping activity of LNA-based HDSSOs targeting the 5′-splice site of *Dmd* exon 23 *in vivo* using *mdx* mice under intramuscular injections

(A) Schematic representation of the SSOs and complementary oligonucleotides used in the study. Each box shows one nucleotide; light blue: LNA; yellow: DNA; dark blue: RNA; red: 2′-OMe RNA. White triangles show phosphorothioate bonds. (B–E) The exon-skipping activity of LNA-based HDSSOs in *mdx* mice. (B and C) Detection of exon 23-skipped dystrophin mRNA in the tibialis anterior muscle of *mdx* 2 weeks after intramuscular injection of SSO or HDSSO at an equimolar dose (B) 2 or (C) 8 nmol. Values represent the mean ± standard error ($n = 4$ per each group). Significant differences compared to the Single were determined using Tukey's test. $^{*}p < 0.05$; $^{***}p < 0.001$; $^{****}p < 0.0001$. (D) Representative images of dystrophin immunostaining in tibialis anterior at 2 weeks after intramuscular injection of SSO or HDSSO. Tibialis anterior from PBS-treated *mdx* and B10 mouse was used as the control sample. Scale bar, 200 μm. (E) Detection of dystrophin restoration by western blot analysis in the tibialis anterior muscle of *mdx* 2 weeks after intramuscular injection of SSO or HDSSO at 2 or 8 nmol compared to *mdx* and WT control B10 mice ($n = 3$ per each group).

We examined the *in vivo* activity of HDSSOs in *mdx* mice under intramuscular injections (Figure 5). As controls, we used both LNA-based HDSSOs with fully PS-linked RNA based complementary oligonucleotides (HDSSOs with full PS) and LNA-based single-stranded SSOs (Single) (Figure 5A). Two weeks after intramuscular injections into each tibialis anterior muscle, exon-skipping activity was analyzed using RT-PCR, immunohistochemistry (IHC), and western blotting analysis. HDSSOs showed higher exon skipping than both HDSSOs with full PS and Single in each tibialis anterior muscle injected at both 2 and 8 nmol (both 8.5 and 34.2 μg as single-stranded LNA-based SSOs) (Figures 5B and 5C). Over 20% of exon 23 mRNA was skipped with the 8-nmol HDSSOs injection. In contrast, HDSSOs with full PS did not induce almost no exon skipping. However, Single induced over 10% of exon 23 skipping. The IHC assay also showed that HDSSOs notably increased dystrophin protein expression compared to tissues from mice treated with single-stranded SSOs (Figure 5D). Furthermore, we also conducted immunoblotting using the same samples and analyzing the dystrophin bands with ImageJ (Fiji) software (Figures 5E and S5).[47] In the tibialis anterior muscle treated with 2 nmol single-stranded SSO, only a slight dystrophin expression was observed. In contrast, the muscle treated with HDSSO exhibited higher dystrophin expression than that of single-stranded SSO. Increasing the dose to 8 nmol, the muscle treated with single-stranded SSO demonstrated higher dystrophin expression than that of 2 nmol single-stranded SSO, whereas the muscle treated with HDSSO showed much higher dystrophin expression than that of single-stranded SSO. At both dosages, the restoration of dystrophin expression by HDSSO injection was significantly superior to that achieved with single-stranded SSO injection.

Figure 5 shows that LNA-based HDSSOs have increased exon-skipping activity *in vivo* following local administration (intramuscular injection). Therefore, we next investigated the *in vivo* activity of HDSSOs in *mdx* mice following systemic subcutaneous (SC)

administration (Figure S6). For systemic SC injections, 11.88 μmol/kg (100 mg/kg as single-stranded LNA-based SSOs) of either HDSSOs or single-stranded SSOs were used. Two weeks after systemic SC injections, we evaluated the exon-skipping activity using RT-PCR (Figure S6B). RT-PCR analyses revealed that HDSSOs did not increase exon-skipping activity compared to single-stranded SSOs. Our group previously reported that lipid-conjugated complementary oligonucleotides enabled an increase in the exon-skipping efficacy of PMO-based HDSSOs under systemic administration.[48] Therefore, we utilized Toc conjugation for the HDSSOs (Figure S6A). Although we also prepared single-stranded SSOs conjugated with Toc, all mice died approximately 30 min after injection (n = 2; data not shown). HDSSOs with Toc conjugation (Toc-HDSSO) exhibited higher exon-skipping activity than single-stranded SSOs without Toc conjugation (Single) and HDSSOs without Toc conjugation (HDSSO), with a statistically significant difference between Toc-HDSSO and Single in the quadriceps.

DISCUSSION

In this study, we uncovered the potential of either DNA- or RNA-based complementary oligonucleotides to increase the exon-skipping activity of LNA-based HDSSOs. Although DNA-based complementary oligonucleotides were generally used for PMO or 2′-OMe RNA-based HDSSOs in previous studies by other groups,[34–37] we found that RNA-based complementary oligonucleotides also seem to be suitable for LNA-based SSOs in HDSSOs (Figure 1). From these results, we hypothesized that different chemistries (DNA or RNA) could be used in complementary oligonucleotides, depending on the chemistries used in SSOs, such as PMO, 2′-OMe RNA, and LNA.

To better understand the appropriate design of complementary oligonucleotides in HDSSOs, we synthesized various complementary oligonucleotides with different chemistries, lengths, and PS linkages (Figure 2). Overall, the results suggest that the design of complementary oligonucleotides is necessary for the efficient exon-skipping activities of LNA-based HDSSOs. It might be effective to distinguish the design of DNA- or RNA-based complementary oligonucleotides because our previous report suggested that the mechanism of HDASOs degradation between DNA-based and RNA-based complementary oligonucleotides seemed to be different.[41] Although the detailed mechanism of decreasing exon-skipping activities by PS linkages is still unknown, our results showed that the RNA-based complementary oligonucleotides allowed many designs compared to DNA-based complementary oligonucleotides, suggesting that RNA-based complementary oligonucleotides are more suitable for LNA-based HDSSOs (Figure 2).

To understand the factors contributing to exon-skipping activity, we monitored the behavior of HDSSOs using time-lapse fluorescence microscopy (Figures 3 and S2). Overall, LNA-based HDSSOs were immediately taken up into the nucleus and taken up more efficiently by nuclei than single-stranded SSOs at 24 h after transfection. In a previous report by Astriab-Fisher et al., complementary oligonucleotides of 2′-OMe RNA-based HDSSOs did not transfer into the nuclei with Lipofectamine 2000.[37] Unlike that report, we used Lipofectamine RNAiMAX to transfect LNA-based HDSSOs into the nucleus, which might explain why the different protocols produced different results. After uptake into the nuclei, the red fluorescence-labeled complementary oligonucleotides disappeared in HDSSOs with some designs, R2, R5, and Wing, which implies that the specific designs of complementary oligonucleotides were digested or excluded from the nuclei (Figure 4). The detailed mechanisms involved, such as whether the complementary oligonucleotides were digested or not, are still unknown. The rate of disappearance of the complementary oligonucleotides also seemed to depend on their design; however, the LNA-based SSOs were stable in the nucleus, regardless of the design of their complementary oligonucleotides. Furthermore, the time-lapse imaging indicated that the extremely high intracellular stability of complementary oligonucleotides negatively affects exon-skipping activity. Indeed, Ono et al. recently reported that RNA-based complementary oligonucleotides in heteroduplex gapmer ASO were more efficiently distributed in the nuclei compared with the single-stranded gapmer ASO and the stability of its complementary oligonucleotides is related to efficient gene silencing.[43] Thus, our results indicated that the distribution of complementary oligonucleotides is also a key factor for efficient splicing modulation in HDSSOs.

We also demonstrated the effectiveness of the HDSSOs *in vivo*. The assay using *mdx* mice under intramuscular injections showed that HDSSOs had increased exon-skipping activity compared to single-stranded SSOs. Our group previously reported that the heteroduplex oligonucleotide (HDO) technology can be applied to both gapmer ASO and anti-microRNAs, which were modified with LNA analogs.[38,49,50] In previous reports, PMO-based SSOs were delivered into targeted myotubes using cDNA oligonucleotides and additional delivery reagents, such as lipofectin and the block co-polymer F127.[35,36] Since PMO does not have a negative charge, DNA-based complementary oligonucleotides were used as negatively charged donors for the delivery reagents. In contrast, this study showed that HDO technology enabled the delivery of LNA-based SSOs into targeted myotubes without the use of additional delivery reagents under intramuscular injection. Therefore, we believe that HDO technologies, using RNA-based complementary oligonucleotides, are promising delivery tools for LNA-based SSOs at least for intramuscular injection.

Recently, several groups have reported that conjugation technologies are efficient delivery strategies for SSOs.[26–29,31,32] The complementary oligonucleotides of HDSSOs can also be used as carriers of conjugates. As Nishina et al. recently noted, direct conjugation with the main strand (gapmer ASO) results in the loss of antisense activity.[38] This means that HDSSOs have two advantages: efficient delivery both *in vitro* and *in vivo* and free conjugation carriers. Our group recently reported that HDO technology with lipid-conjugated cRNA enables an increase in the exon-skipping efficacy of PMO-based SSOs *in vivo* under systemic injections.[48] In this study, we revealed that Toc conjugation into RNA-based complementary oligonucleotides increased the exon-skipping efficacy of LNA-based HDSSOs, with a statistically

significant difference between Toc-conjugated HDSSO and single-stranded LNA-based SSO in the quadriceps under systemic SC injection (Figure S6). Another group recently reported prospective peptides for the efficient delivery of SSOs targeting *DMD* mRNA.[29] We expect that these conjugates could be delivered by complementary oligonucleotides in HDSSOs without losing the exon-skipping activity of SSOs.

The present study also indicates that HDO technology reduces the toxicity of Toc-conjugated single-stranded LNA-based SSOs (Figure S6). Thus, mice injected with Toc-conjugated single-stranded LNA-based SSOs died approximately 30 min after systemic SC injection, but mice injected with Toc-conjugated HDSSOs survived. Although a recent publication indicated the LNA analogs enhance the exon-skipping activity of SSO,[51] LNA-based SSOs induce unintended splicing alterations.[52] Furthermore, our group previously reported that LNA-based gapmer ASO with cholesterol conjugation showed lethal toxicities.[53] In the future, we hope to investigate the off-target effects of LNA-based SSOs using HDO technology with either lipid-conjugation or peptide conjugation.

The LNA-based SSOs in the present study were selected from the optimized designed SSOs in our previous study, which target the skipping of exon 58 in the human dystrophin gene.[10] Although the targetable genetic abnormalities of the SSOs are very rare in DMD,[54] these SSOs have the potential to be a promising treatment of DMD patients, including personalized n-of-1 therapy. However, there is a methodological limitation in our analysis of exon 58 skipping because we only evaluated skipping activity using RT-qPCR for a single exon 58 and not in myogenic and HEK293 cells. RT-PCR analyses including several exons flanking exon 58 and myogenic cells would yield a more accurate assessment for estimating the skipping activity of SSOs.

In conclusion, we found that HDSSOs represent a prospective strategy for increasing exon-skipping activity both *in vitro* and *in vivo*. Although the detailed mechanism involved is still unknown, HDSSOs could be one of the options for efficient SSO delivery. We also reported that the optimization of complementary oligonucleotides, including factors such as chemistry and the PS backbone, is important for higher exon-skipping activity.

MATERIALS AND METHODS

Synthesis of oligonucleotides

All SSOs and complementary oligonucleotides used in this study are listed in Tables S1–S7. Chemical modifications, such as LNA and 2′-OMe RNA, were used for the SSO sequences, where the phosphodiester linkages were completely replaced by PS linkages. LNA or 2′-OMe RNA-based SSOs designed to have sequences complementary to those of human *DMD* were synthesized and purified by GeneDesign (Osaka, Japan). For complementary oligonucleotides, chemical modifications such as LNA, 2′-OMe RNA, and PS linkages were used on a case-by-case basis. Complementary oligonucleotides were designed with sequences complementary to each SSO.

All DNA primers used in this study are listed in Table S8, the figure legend of Figure S4, and the section "estimation of skipping efficiency in stable cell line." DNA primers were synthesized and purified by Hokkaido System Sciences (Hokkaido, Japan) and Eurofins Genomics K.K. (Tokyo, Japan).

Annealing oligonucleotides for HDSSOs

To prepare HDSSOs, we annealed the SSOs and complementary oligonucleotides. For *in vitro* study, each SSO and complementary oligonucleotide was dissolved in 50 mM Tris buffer (pH 7.5) containing 100 mM NaCl to a final concentration of 4 μM. For Figure 1, the dissolved HDSSOs were boiled and followed by slow cooling to room temperature for 8 h. For Figures 2, 3, and 4, the dissolved HDSSOs were denatured at 99°C for 5 min. Then, after incubating at 95°C for 20 min, the dissolved HDSSOs were cooled to room temperature for 3.5 h.

For *in vivo* study, each SSO and complementary oligonucleotide was dissolved in PBS to a final concentration of 500 μM. The dissolved HDSSOs were annealed at 95°C for 5 min and then incubated at 37°C for 1 h.

Estimation of skipping efficiency in stable cell line

The Flp-In 293 cells (Thermo Fisher Scientific, Waltham, MA)-based stable reporter cells[10] were used for the assay. Briefly, the stable cells encode the human DMD minigene, which contains exon 58 and its adjacent introns. In the minigene, the intron 57 was shortened to remove it from position +207 to +17,486 because its length is over 17 kbp. The stable cells were cultured in high-glucose Dulbecco's Modified Eagle Medium (DMEM) containing 10% fetal bovine serum (FBS) (Biowest, Nuaillé, France), 1× antibiotic-antimycotic (AA) solution for cell culture (Sigma-Aldrich, St. Louis, MO), and 100 μg/mL hygromycin (Thermo Fisher Scientific) and maintained in a 5% CO_2 incubator at 37°C.

Stable reporter cells were seeded 1 day before SSO transfection at a density of 80,000 (Figure 1) or 200,000 (Figure 2) cells/well in 24-well plates (Iwaki, Tokyo, Japan). After 24 h, the cells were transfected with SSOs at a concentration of 10 nM (Figure 1) or 30 nM (Figure 2) using Lipofectamine RNAiMAX, according to the manufacturer's protocols, and grown in high-glucose DMEM containing 10% FBS and 1× AA. Twenty-four hours after HDSSOs transfection, the cells were harvested and used for assays.

At 24 h after transfection, total RNA was isolated from the samples using QuickGene 800 (Kurabo, Osaka, Japan), QuickGene RNA cultured cell kit S (Kurabo), and RQ1 RNase-Free DNase (Promega, Fitchburg, WI) according to the manufacturer's instructions. Total RNA was reverse transcribed using ReverTra Ace qPCR RT Master Mix (Toyobo, Osaka, Japan) according to the manufacturer's instructions. cDNA dilution was used as the template for individual PCR reactions using specific primer sets (Table S8) designed using the Primer BLAST program.[55] RT-qPCR was performed using StepOnePlus Real-Time PCR System (Thermo Fisher Scientific)

and SYBR Green Real-Time PCR Master Mix Plus (Toyobo) according to the manufacturers' protocols, except that the annealing condition was set at 65°C for 15 s. The DNA primers targeting *DMD* exon 58-skipped mRNA were used as previously described.[20,56] The expression levels of human *GAPDH* or human *RPLP2* mRNA were used to normalize the data. Each experiment was repeated three times to ensure the reproducibility of results.

UV melting analysis

The T_m value of each HDSSO was measured, as reported in our previous study.[10] Briefly, each SSO and a complementary strand were dissolved in 10 mM sodium phosphate buffer (pH 7.2) containing 10 mM NaCl to a final concentration of 2 μM. The samples were boiled and followed by slow cooling to room temperature for 8 h. The absorbance at 260 nm was measured from 5°C to 95°C at a scan rate of 0.5°C/min. The peak temperature in the derivative curve was determined to be the T_m. The experiment was repeated three or four times to ensure the reproducibility of the results.

Time-lapse microscopy imaging

Because the stable cells used for exon-skipping activity evaluation also encode the fluorescent proteins EGFP and DsRed,[10] we selected HEK293 cells for time-lapse microscopy imaging. HEK293 cells were cultured in high-glucose DMEM containing 10% FBS and 1× AA and maintained in a 5% CO_2 incubator at 37°C. Twenty-four hours before transfection, HEK293 cells were seeded at 24,000 cells/well on Collagen I Cellware 96-Well Black/Clear Plates (Becton Dickinson Labware, Franklin Lakes, NJ) in 100 μL/well FluoroBrite DMEM (Gibco, Waltham, MA) containing 10% FBS, 1× AA solution, and 1× GlutaMAX Supplement (Thermo Fisher Scientific). Fifteen minutes before transfection, the cells were suspended in FluoroBrite DMEM containing 10% FBS, 1× AA solution, 1× GlutaMAX Supplement, and 0.1 μg/mL Hoechst 33342 (Lonza, Walkersville, MD) to image the nucleus. After HDSSO transfection at a concentration of 10 nM using Lipofectamine RNAiMAX, the cells were analyzed using a BZ-X700 microscope (Keyence, Osaka, Japan) with an incubation system that stably incubated the cells at 37°C in humidified 5% CO_2. Phase-contrast and fluorescence images were obtained at a 20× magnification. Fluorescence images were collected using excitation wavelength/emission wavelength 360/460-, 470/525-, and 620/700-nm filters (Keyence) for Hoechst 33342, 6-FAM, and Alexa 647 staining, respectively. Images were captured every 30 min for 24 h post-HDSSOs transfection. Image processing (e.g., black balance, merging) was performed using BZ-X Analyzer software (Keyence). The time-lapse videos of merged images were generated using ImageJ (Fiji)[47] and shown in Videos S1A, S1B, S1C, S1D, S1E, S1F, and S1G. The images per sample were captured from a single field of view, and the experiments were repeated twice to ensure reproducibility of the results (n = 2, Singlet).

Animal experiments

Mdx mice (*mdx*, C57BL/10ScSc-Dmdmdx/J, males) were obtained from CLEA Japan (Tokyo, Japan) and bred. Prior to postmortem analyses, mice were anesthetized by inhalation with 4% isoflurane in a draft and sacrificed by cervical dislocation after blood collection. All protocols met ethics and safety guidelines for animal experimentation and were approved by the ethics committee of Tokyo Medical and Dental University (no. A2021-191C and A2023-144C2).

Estimation of skipping efficiency *in vivo*

LNA-based SSOs and complementary oligonucleotides were designed based on previous reports (Table S7).[44–46] For the *in vivo* study, 5- to 6-week-old *mdx* mice were used. Each HDSSO targeting exon 23 of the dystrophin gene was dissolved in PBS. HDSSOs or SSOs (2 or 8 nmol) were injected into the tibialis anterior muscle or 11.88 μmol/kg of each SSO was injected SC. Two weeks after injection, the mice were sacrificed under anesthesia with 4% isoflurane (Wako, Osaka, Japan), and muscles were dissected. Muscle samples for RT-PCR were placed in tubes and frozen in liquid nitrogen, while muscle samples for IHC were immediately snap-frozen in liquid nitrogen-cooled isopentane.

Total RNA was extracted from muscle tissues using Isogen 2 (Nippon Gene, Tokyo, Japan), and 500 ng total RNA was used for one-step RT-PCR (Qiagen, Venlo, the Netherlands) according to the manufacturers' instructions. The primer sequences were mEx22F 5′-ATC CAGCAGTCAGAAAGCAAA-3′ and mEx24R 5′-CAGCCATC CATTTCTGTAAGG-3′ for the amplification of exons 22 to 24. The PCR conditions were 50°C for 30 min and 95°C for 15 min, then 35 cycles of 94°C for 1 min, 60°C for 1 min, 72°C for 1 min, and 72°C for 7 min. The intensity of the PCR bands was analyzed using Bioanalyzer 2100 (Agilent, Santa Clara, CA), and skipping efficiency was calculated using the following formula: (intensity of skipped band)/(intensity of skipped band + intensity of unskipped band). The resulting PCR bands were extracted using a gel extraction kit for direct sequencing to confirm exon skipping.

IHC

Ten-micrometer cryosections were cut from flash-frozen muscle, placed on Platinum Pro coated slides (Matsunami Glass Industrial, Osaka, Japan), and air-dried. The sections were stained with monoclonal rabbit antibody ab15277 (Abcam, Cambridge, UK) against the C terminus of muscular dystrophin and anti-rabbit goat conjugated with Alexa Fluor 568 (Thermo Fisher Scientific) as a secondary antibody. For nuclear counterstaining, 4′,6-diamidino-2-phenylindole containing a mounting agent was used.

Western blotting

Proteins were extracted from sliced frozen muscle using SDS buffer (0.125 M Tris/HCl at pH 6.4, 10% glycerol, 4% SDS, 4 M urea, 10% β-mercaptoethanol, and 0.005% bromophenol blue) supplemented with 1× protease inhibitor (Complete Mini, Roche Diagnostics, Mannheim, Germany). The normal control lysate from C57BL/10ScNJic (B10) mice was also prepared as a reference for dystrophin expression. Subject and normal control lysates were denatured at 100°C for 3 min and electrophoresed on 4%–15% gradient polyacrylamide gel (Bio-Rad, Hercules, CA) at 120 V for 90 min. The proteins were then transferred to a polyvinylidene fluoride membrane (Bio-Rad)

by wet transfer at 30 V overnight. After incubation with 5% nonfat milk in Tris-buffered saline with 0.1% Tween 20 Detergent (TBS-T) for 60 min, the membrane was incubated at 4°C overnight with an anti-dystrophin antibody (1:200) (ab15277, Abcam) or anti-vinculin antibody (1:1,000) (NB600-1293, Novus Biologicals, Centennial, CO). The membrane was washed 3 times for 10 min in TBS-T and incubated with a 1:10,000 dilution of horseradish peroxidase-conjugated anti-rabbit or anti-mouse antibodies (Jackson Immuno Research, West Grove, PA) for 60 min. The membrane was washed three times with TBS-T and then developed with West Dura Extended Duration Substrate (Thermo Fisher Scientific) according to the manufacturers' protocols. The immunoreactive bands were detected by ChemiDoc Image System (Bio-Rad).

Statistical analysis

The data shown in Figures 1, 2, and S5 are expressed as mean ± standard deviation (SD). Statistical analysis for Figure 2 was performed using one-way analysis of variance (ANOVA) with Dunnett's T3 test for multiple comparisons with unequal variances. R software (version 4.0.3), R-Studio (version 1.4.1103), and the R package PMCMRplus (dunnettT3Test mode) were used for statistical analyses.[57–59]

The data shown in Figures 5 and S6 are expressed as mean ± standard error. Statistical analyses were performed in GraphPad Prism version 9 using ANOVA with Tukey's test.

DATA AVAILABILITY

The datasets analyzed in the present study are available from the corresponding author upon reasonable request.

ACKNOWLEDGMENTS

T.S. was supported by Grant-in-aid for JSPS research fellow under grant no. 15J05689. K.T. was supported by the Project Meet and Osaka University. S.O. was supported by the Japan Agency for Medical Research and Development under grant nos. JP15am0301004 and JP19am0401003.

AUTHOR CONTRIBUTIONS

T.S., J.H., Y.N., K.A., and T.N. conducted the experiments. T.S., J.H., K.Y., T.N., K.T., T.Y., and S.O. designed the experiments and wrote the paper.

DECLARATION OF INTERESTS

T.Y. collaborates with Takeda Pharmaceuticals, Daiichi Sankyo, Rena Therapeutics, Toray Industries, Eisai, and Sumitomo Pharma, and serves as the academic adviser for Rena Therapeutics and Braizon Therapeutics. Patents WO2014203518A1 related to this paper have been filed by T.Y., K.Y., S.O., and T.S.

SUPPLEMENTAL INFORMATION

Supplemental information can be found online at https://doi.org/10.1016/j.omtn.2025.102468.

REFERENCES

1. Stein, C.A., and Castanotto, D. (2017). FDA-Approved Oligonucleotide Therapies in 2017. Mol. Ther. *25*, 1069–1075.
2. Corey, D.R. (2017). Nusinersen, an antisense oligonucleotide drug for spinal muscular atrophy. Nat. Neurosci. *20*, 497–499.
3. Duan, D., Goemans, N., Takeda, S., Mercuri, E., and Aartsma-Rus, A. (2021). Duchenne muscular dystrophy. Nat. Rev. Dis. Prim. *7*, 13.
4. Kim, J., Hu, C., Moufawad El Achkar, C., Black, L.E., Douville, J., Larson, A., Pendergast, M.K., Goldkind, S.F., Lee, E.A., Kuniholm, A., et al. (2019). Patient-Customized Oligonucleotide Therapy for a Rare Genetic Disease. N. Engl. J. Med. *381*, 1644–1652.
5. Synofzik, M., van Roon-Mom, W.M.C., Marckmann, G., van Duyvenvoorde, H.A., Graessner, H., Schüle, R., and Aartsma-Rus, A. (2022). Preparing n-of-1 Antisense Oligonucleotide Treatments for Rare Neurological Diseases in Europe: Genetic, Regulatory, and Ethical Perspectives. Nucleic Acid Therapeut. *32*, 83–94.
6. Kim, J., Woo, S., de Gusmao, C.M., Zhao, B., Chin, D.H., DiDonato, R.L., Nguyen, M.A., Nakayama, T., Hu, C.A., Soucy, A., et al. (2023). A framework for individualized splice-switching oligonucleotide therapy. Nature *619*, 828–836.
7. Dominski, Z., and Kole, R. (1993). Restoration of correct splicing in thalassemic pre-mRNA by antisense oligonucleotides. Proc. Natl. Acad. Sci. USA *90*, 8673–8677.
8. Wilton, S.D., Fall, A.M., Harding, P.L., McClorey, G., Coleman, C., and Fletcher, S. (2007). Antisense oligonucleotide-induced exon skipping across the human dystrophin gene transcript. Mol. Ther. *15*, 1288–1296.
9. Aartsma-Rus, A., van Vliet, L., Hirschi, M., Janson, A.A.M., Heemskerk, H., de Winter, C.L., de Kimpe, S., van Deutekom, J.C.T., t Hoen, P.A.C., and van Ommen, G.-J.B. (2009). Guidelines for Antisense Oligonucleotide Design and Insight Into Splice-modulating Mechanisms. Mol. Ther. *17*, 548–553.
10. Shimo, T., Tachibana, K., Saito, K., Yoshida, T., Tomita, E., Waki, R., Yamamoto, T., Doi, T., Inoue, T., Kawakami, J., and Obika, S. (2014). Design and evaluation of locked nucleic acid-based splice-switching oligonucleotides *in vitro*. Nucleic Acids Res. *42*, 8174–8187.
11. Disterer, P., Kryczka, A., Liu, Y., Badi, Y.E., Wong, J.J., Owen, J.S., and Khoo, B. (2014). Development of therapeutic splice-switching oligonucleotides. Hum. Gene Ther. *25*, 587–598.
12. Shimo, T., Maruyama, R., and Yokota, T. (2018). Designing Effective Antisense Oligonucleotides for Exon Skipping. Methods Mol. Biol. *1687*, 143–155.
13. Aartsma-Rus, A., Kaman, W.E., Bremmer-Bout, M., Janson, A.A.M., den Dunnen, J.T., van Ommen, G.J.B., and van Deutekom, J.C.T. (2004). Comparative analysis of antisense oligonucleotide analogs for targeted DMD exon 46 skipping in muscle cells. Gene Ther. *11*, 1391–1398.
14. Roberts, J., Palma, E., Sazani, P., Ørum, H., Cho, M., and Kole, R. (2006). Efficient and persistent splice switching by systemically delivered LNA oligonucleotides in mice. Mol. Ther. *14*, 471–475.
15. Guterstam, P., Lindgren, M., Johansson, H., Tedebark, U., Wengel, J., El Andaloussi, S., and Langel, U. (2008). Splice-switching efficiency and specificity for oligonucleotides with locked nucleic acid monomers. Biochem. J. *412*, 307–313.
16. Graziewicz, M.A., Tarrant, T.K., Buckley, B., Roberts, J., Fulton, L., Hansen, H., Ørum, H., Kole, R., and Sazani, P. (2008). An Endogenous TNF-α Antagonist Induced by Splice-switching Oligonucleotides Reduces Inflammation in Hepatitis and Arthritis Mouse Models. Mol. Ther. *16*, 1316–1322.
17. Le, B.T., Murayama, K., Shabanpoor, F., Asanuma, H., and Veedu, R.N. (2017). Antisense oligonucleotide modified with serinol nucleic acid (SNA) induces exon skipping in mdx myotubes. RSC Adv. *7*, 34049–34052.
18. Le, B.T., Adams, A.M., Fletcher, S., Wilton, S.D., and Veedu, R.N. (2017). Rational Design of Short Locked Nucleic Acid-Modified 2′-O-Methyl Antisense Oligonucleotides for Efficient Exon-Skipping In Vitro. Mol. Ther. Nucleic Acids *9*, 155–161.
19. Shimo, T., Tachibana, K., and Obika, S. (2018). Construction of a tri-chromatic reporter cell line for the rapid and simple screening of splice-switching oligonucleotides targeting DMD exon 51 using high content screening. PLoS One *13*, e0197373.
20. Shimo, T., Tachibana, K., Kawawaki, Y., Watahiki, Y., Ishigaki, T., Nakatsuji, Y., Hara, T., Kawakami, J., and Obika, S. (2019). Enhancement of exon skipping activity by reduction in the secondary structure content of LNA-based splice-switching oligonucleotides. Chem. Commun. *55*, 6850–6853.

21. Masaki, Y., Yamamoto, K., Inde, T., Yoshida, K., Maruyama, A., Nagata, T., Tanihata, J., Takeda, S., Sekine, M., and Seio, K. (2019). Synthesis of 2′-O-(N-methylcarbamoylethyl) 5-methyl-2-thiouridine and its application to splice-switching oligonucleotides. Bioorg. Med. Chem. Lett. *29*, 160–163.

22. Chen, S., Le, B.T., Chakravarthy, M., Kosbar, T.R., and Veedu, R.N. (2019). Systematic evaluation of 2'-Fluoro modified chimeric antisense oligonucleotide-mediated exon skipping *in vitro*. Sci. Rep. *9*, 6078.

23. Shimo, T., Nakatsuji, Y., Tachibana, K., and Obika, S. (2021). Design and In Vitro Evaluation of Splice-Switching Oligonucleotides Bearing Locked Nucleic Acids, Amido-Bridged Nucleic Acids, and Guanidine-Bridged Nucleic Acids. Int. J. Mol. Sci. *22*, 3526.

24. Baker, Y.R., Thorpe, C., Chen, J., Poller, L.M., Cox, L., Kumar, P., Lim, W.F., Lie, L., McClorey, G., Epple, S., et al. (2022). An LNA-amide modification that enhances the cell uptake and activity of phosphorothioate exon-skipping oligonucleotides. Nat. Commun. *13*, 4036.

25. Nakagawa, O., Ming, X., Huang, L., and Juliano, R.L. (2010). Targeted intracellular delivery of antisense oligonucleotides via conjugation with small-molecule ligands. J. Am. Chem. Soc. *132*, 8848–8849.

26. Betts, C., Saleh, A.F., Arzumanov, A.A., Hammond, S.M., Godfrey, C., Coursindel, T., Gait, M.J., and Wood, M.J. (2012). Pip6-PMO, A New Generation of Peptide-oligonucleotide Conjugates With Improved Cardiac Exon Skipping Activity for DMD Treatment. Mol. Ther. Nucleic Acids *1*, e38.

27. Shabanpoor, F., and Gait, M.J. (2013). Development of a general methodology for labelling peptide-morpholino oligonucleotide conjugates using alkyne-azide click chemistry. Chem. Commun. *49*, 10260–10262.

28. Shabanpoor, F., McClorey, G., Saleh, A.F., Järver, P., Wood, M.J.A., and Gait, M.J. (2015). Bi-specific splice-switching PMO oligonucleotides conjugated via a single peptide active in a mouse model of Duchenne muscular dystrophy. Nucleic Acids Res. *43*, 29–39.

29. Jirka, S.M.G., t Hoen, P.A.C., Diaz Parillas, V., Tanganyika-de Winter, C.L., Verheul, R.C., Aguilera, B., de Visser, P.C., and Aartsma-Rus, A.M. (2018). Cyclic Peptides to Improve Delivery and Exon Skipping of Antisense Oligonucleotides in a Mouse Model for Duchenne Muscular Dystrophy. Mol. Ther. *26*, 132–147.

30. Hande, M., Saher, O., Lundin, K.E., Smith, C.I.E., Zain, R., and Lönnberg, T. (2019). Oligonucleotide(-)Palladacycle Conjugates as Splice-Correcting Agents. Molecules *24*, 1180.

31. Tone, Y., Mamchaoui, K., Tsoumpra, M.K., Hashimoto, Y., Terada, R., Maruyama, R., Gait, M.J., Arzumanov, A.A., McClorey, G., Imamura, M., et al. (2021). Immortalized Canine Dystrophic Myoblast Cell Lines for Development of Peptide-Conjugated Splice-Switching Oligonucleotides. Nucleic Acid Therapeut. *31*, 172–181.

32. Lim, K.R.Q., Woo, S., Melo, D., Huang, Y., Dzierlega, K., Shah, M.N.A., Aslesh, T., Roshmi, R.R., Echigoya, Y., Maruyama, R., et al. (2022). Development of DG9 peptide-conjugated single- and multi-exon skipping therapies for the treatment of Duchenne muscular dystrophy. Proc. Natl. Acad. Sci. USA *119*, e2112546119.

33. Gait, M.J., Arzumanov, A.A., McClorey, G., Godfrey, C., Betts, C., Hammond, S., and Wood, M.J.A. (2019). Cell-Penetrating Peptide Conjugates of Steric Blocking Oligonucleotides as Therapeutics for Neuromuscular Diseases from a Historical Perspective to Current Prospects of Treatment. Nucleic Acid Therapeut. *29*, 1–12.

34. Morcos, P.A. (2001). Achieving efficient delivery of morpholino oligos in cultured cells. Genesis *30*, 94–102.

35. Gebski, B.L., Mann, C.J., Fletcher, S., and Wilton, S.D. (2003). Morpholino antisense oligonucleotide induced dystrophin exon 23 skipping in mdx mouse muscle. Hum. Mol. Genet. *12*, 1801–1811.

36. Fletcher, S., Honeyman, K., Fall, A.M., Harding, P.L., Johnsen, R.D., and Wilton, S.D. (2006). Dystrophin expression in the mdx mouse after localised and systemic administration of a morpholino antisense oligonucleotide. J. Gene Med. *8*, 207–216.

37. Astriab-Fisher, A., Fisher, M.H., Juliano, R., and Herdewijn, P. (2004). Increased uptake of antisense oligonucleotides by delivery as double stranded complexes. Biochem. Pharmacol. *68*, 403–407.

38. Nishina, K., Piao, W., Yoshida-Tanaka, K., Sujino, Y., Nishina, T., Yamamoto, T., Nitta, K., Yoshioka, K., Kuwahara, H., Yasuhara, H., et al. (2015). DNA/RNA heteroduplex oligonucleotide for highly efficient gene silencing. Nat. Commun. *6*, 7969.

39. Nishina, T., Numata, J., Nishina, K., Yoshida-Tanaka, K., Nitta, K., Piao, W., Iwata, R., Ito, S., Kuwahara, H., Wada, T., et al. (2015). Chimeric Antisense Oligonucleotide Conjugated to alpha-Tocopherol. Mol. Ther. Nucleic Acids *4*, e220.

40. Nagata, T., Dwyer, C.A., Yoshida-Tanaka, K., Ihara, K., Ohyagi, M., Kaburagi, H., Miyata, H., Ebihara, S., Yoshioka, K., Ishii, T., et al. (2021). Cholesterol-functionalized DNA/RNA heteroduplexes cross the blood-brain barrier and knock down genes in the rodent CNS. Nat. Biotechnol. *39*, 1529–1536.

41. Asami, Y., Nagata, T., Yoshioka, K., Kunieda, T., Yoshida-Tanaka, K., Bennett, C.F., Seth, P.P., and Yokota, T. (2021). Efficient Gene Suppression by DNA/DNA Double-Stranded Oligonucleotide In Vivo. Mol. Ther. *29*, 838–847.

42. Watkins, N.E., Jr., and SantaLucia, J., Jr. (2005). Nearest-neighbor thermodynamics of deoxyinosine pairs in DNA duplexes. Nucleic Acids Res. *33*, 6258–6267.

43. Ono, D., Asada, K., Yui, D., Sakaue, F., Yoshioka, K., Nagata, T., and Yokota, T. (2021). Separation-related rapid nuclear transport of DNA/RNA heteroduplex oligonucleotide: unveiling distinctive intracellular trafficking. Mol. Ther. Nucleic Acids *23*, 1360–1370.

44. Wilton, S.D., Lloyd, F., Carville, K., Fletcher, S., Honeyman, K., Agrawal, S., and Kole, R. (1999). Specific removal of the nonsense mutation from the mdx dystrophin mRNA using antisense oligonucleotides. Neuromuscul. Disord. *9*, 330–338.

45. Mann, C.J., Honeyman, K., Cheng, A.J., Ly, T., Lloyd, F., Fletcher, S., Morgan, J.E., Partridge, T.A., and Wilton, S.D. (2001). Antisense-induced exon skipping and synthesis of dystrophin in the mdx mouse. Proc. Natl. Acad. Sci. USA *98*, 42–47.

46. Harding, P.L., Fall, A.M., Honeyman, K., Fletcher, S., and Wilton, S.D. (2007). The influence of antisense oligonucleotide length on dystrophin exon skipping. Mol. Ther. *15*, 157–166.

47. Schindelin, J., Arganda-Carreras, I., Frise, E., Kaynig, V., Longair, M., Pietzsch, T., Preibisch, S., Rueden, C., Saalfeld, S., Schmid, B., et al. (2012). Fiji: an open-source platform for biological-image analysis. Nat. Methods *9*, 676–682.

48. Hasegawa, J., Nagata, T., Ihara, K., Tanihata, J., Ebihara, S., Yoshida-Tanaka, K., Yanagidaira, M., Ohara, M., Sasaki, A., Nakayama, M., et al. (2024). Heteroduplex oligonucleotide technology boosts oligonucleotide splice switching activity of morpholino oligomers in a Duchenne muscular dystrophy mouse model. Nat. Commun. *15*, 7530.

49. Yoshioka, K., Kunieda, T., Asami, Y., Guo, H., Miyata, H., Yoshida-Tanaka, K., Sujino, Y., Piao, W., Kuwahara, H., Nishina, K., et al. (2019). Highly efficient silencing of microRNA by heteroduplex oligonucleotides. Nucleic Acids Res. *47*, 7321–7332.

50. Suzuki, M., Ishibashi, S., Iwasawa, E., Oguma, T., Saito, Y., Li, F., Otsu, S., Ichinose, K., Yoshioka, K., Nagata, T., and Yokota, T. (2021). Effective silencing of miR-126 after ischemic stroke by means of intravenous alpha-tocopherol-conjugated heteroduplex oligonucleotide in mice. Sci. Rep. *11*, 14237.

51. van Deutekom, J., Beekman, C., Bijl, S., Bosgra, S., van den Eijnde, R., Franken, D., Groenendaal, B., Harquouli, B., Janson, A., Koevoets, P., et al. (2023). Next Generation Exon 51 Skipping Antisense Oligonucleotides for Duchenne Muscular Dystrophy. Nucleic Acid Therapeut. *33*, 193–208.

52. Zaw, K., Greer, K., Aung-Htut, M.T., Mitrpant, C., Veedu, R.N., Fletcher, S., and Wilton, S.D. (2019). Consequences of Making the Inactive Active Through Changes in Antisense Oligonucleotide Chemistries. Front. Genet. *10*, 1249.

53. Wada, S., Yasuhara, H., Wada, F., Sawamura, M., Waki, R., Yamamoto, T., Harada-Shiba, M., and Obika, S. (2016). Evaluation of the effects of chemically different linkers on hepatic accumulations, cell tropism and gene silencing ability of cholesterol-conjugated antisense oligonucleotides. J. Contr. Release *226*, 57–65.

54. Aartsma-Rus, A., Fokkema, I., Verschuuren, J., Ginjaar, I., van Deutekom, J., van Ommen, G.J., and den Dunnen, J.T. (2009). Theoretic applicability of antisense-mediated exon skipping for Duchenne muscular dystrophy mutations. Hum. Mutat. *30*, 293–299.

55. Ye, J., Coulouris, G., Zaretskaya, I., Cutcutache, I., Rozen, S., and Madden, T.L. (2012). Primer-BLAST: a tool to design target-specific primers for polymerase chain reaction. BMC Bioinf. *13*, 134.

56. Shimo, T., Hosoki, K., Nakatsuji, Y., Yokota, T., and Obika, S. (2018). A novel human muscle cell model of Duchenne muscular dystrophy created by CRISPR/Cas9 and evaluation of antisense-mediated exon skipping. J. Hum. Genet. *63*, 365–375.

57. Team, R.C. (2020). R: A Language and Environment for Statistical Computing (Vienna, Austria: R Foundation for Statistical Computing).

58. Team, R. (2020). RStudio: Integrated Development for R (Boston, MA: RStudio, PBC). http://wwwrstudiocom/.

59. Pohlert, T. (2020). PMCMRplus: calculate pairwise multiple comparisons of mean rank sums. https://CRANR-projectorg/package=PMCMRplus.

OMTN, Volume *36*

Supplemental information

Effect of chemical modification

on the exon-skipping activity

# of heteroduplex oligonucleotides

Takenori Shimo, Juri Hasegawa, Kotaro Yoshioka, Yusuke Nakatsuji, Kotomi Aso, Keisuke Tachibana, Tetsuya Nagata, Takanori Yokota, and Satoshi Obika

Table S1 Complementary oligonucleotides used for Figure 1.
Complementary oligonucleotides for *DMD* gene exon 58 skipping are shown. Sequences are shown from 5′ to 3′. Uppercase letters: RNA. Lowercase letters: DNA. Uppercase letters with bold and italic: 2′-OMe RNA. ^: phosphorothioate.

Entry	Name	Sequence
01	Wing	***G***^***G***^CCUUCAAGAGG***G***^***A***
02	DNA	ggccttcaagaggga
03	RNA	GGCCUUCAAGAGGGA

Table S2 SSOs used for Figures 1 and 2.

LNA-based SSOs and 2'-OMe RNA based SSOs for *DMD* gene exon 58 skipping are shown. Sequences are shown from 5′ to 3′. Lowercase letter: DNA, uppercase letter with bold and underline: LNA, **C**: LNA-5-methylcytosine, uppercase letters with bold and italic: 2′-OMe RNA, and ^: phosphorothioate.

Entry	Name	Sequence
01	LNA based SSO	t^c^**C**^c^t^**C**^t^t^**G**^a^a^**G**^g^c^**C**
02	2'-OMe based SSO	***U^C^C^C^U^C^U^U^G^A^A^G^G^C^C***

Table S3 Complementary oligonucleotides used for FigureS1.
Complementary oligonucleotides for *DMD* gene exon 58 skipping are shown. Sequences are shown from 5′ to 3′. Uppercase letters: RNA. Lowercase letters: DNA. Uppercase letters with bold and italic: 2′-OMe RNA. ^: phosphorothioate.

Entry	Name	Sequence
01	Wing	***C***^***C***^AGGAGCCCAGA***G***^***G***
02	DNA	Ccaggagcccagagg
03	RNA	CCAGGAGCCCAGAGG

Table S4 SSOs used for FigureS1.

LNA-based SSOs and 2'-OMe RNA based SSOs for *DMD* gene exon 58 skipping are shown. Sequences are shown from 5′ to 3′. Lowercase letter: DNA, uppercase letter with bold and underline: LNA, **C**: LNA-5-methylcytosine, uppercase letters with bold and italic: 2′-OMe RNA, and ^: phosphorothioate.

Entry	Name	Sequence
01	LNA based SSO	**C**^c^t^**C**^t^g^**G**^g^c^**T**^c^c^**T**^g^g
02	2'-OMe based SSO	***C***^***C***^***U***^***C***^***U***^***G***^***G***^***G***^***C***^***U***^***C***^***C***^***U***^***G***^***G***

Table S5 Complementary oligonucleotides used for the assay.
Complementary oligonucleotides for *DMD* gene exon 58 skipping are shown. Sequences are shown from 5′ to 3′. Lowercase letter: DNA, uppercase letter: RNA, uppercase letter with bold and underline: LNA, uppercase letters with bold and italic: 2′-OMe RNA, i: 2′-deoxyinosine, **I**: inosine, and ^: phosphorothioate.

Entry	Name	Sequence
1	D1	acaggccttcaagagggaatt
2	D2	ggccttcaagaggga
	(also called “DNA” in Fig.1)	
3	D3	cttcaagag
4	D4	g^g^c^c^t^t^c^a^a^g^a^g^g^g^a
5	D5	g^g^ccttcaagaggg^a
6	D6	**G**^**G**^ccttcaagagg**G**^**A**
7	D7	iiccttcaaiaiiia
8	R1	ACAGGCCUUCAAGAGGGAAUU
9	R2	GGCCUUCAAGAGGGA
	(also called “RNA” in Fig.1)	
10	R3	CUUCAAGAG
11	R4	G^G^C^C^U^U^C^A^A^G^A^G^G^G^A
12	R5	G^G^CCUUCAAGAGGG^A
13	R6	**G**^**G**^CCUUCAAGAGG**G**^**A**
14	R7	IICCUUCAAIAIIIA
15	Wing	***G***^***G***^CCUUCAAGAGG***G***^***A***

Table S6 SSOs and complementary oligonucleotides used for the time-lapse microscopy imaging.
LNA-based SSOs and 2'-OMe RNA based SSOs for *DMD* gene exon 58 skipping and their complementary oligonucleotides are shown. Sequences are shown from 5′ to 3′. Lowercase letter: DNA, uppercase letter: RNA, uppercase letter with bold and underline: LNA, <u>**C**</u>: LNA-5-methylcytosine, uppercase letters with bold and italic: 2′-OMe RNA, f: 6-FAM, Y_NHC6: Alexa-647 with an aminohexyl linker and ^: phosphorothioate.

Entry	Name	Sequence
01	f_DMDexon58-1+14_5/15	f^t^c^<u>**C**</u>^c^t^<u>**C**</u>^t^t^<u>**G**</u>^a^a^<u>**G**</u>^g^c^<u>**C**</u>
02	Y_wing	Y_NHC6^***G***^***G***^CCUUCAAGAGG***G***^***A***
03	Y_R2	Y_NHC6^GGCCUUCAAGAGGGA
04	Y_R4	Y_NHC6^G^G^C^C^U^U^C^A^A^G^A^G^G^G^A
05	Y_R5	Y_NHC6^G^G^CCUUCAAGAGGG^A
06	Y_R6	Y_NHC6^<u>**G**</u>^<u>**G**</u>^CCUUCAAGAGG<u>**G**</u>^<u>**A**</u>

Table S7 SSOs and complementary oligonucleotides used for murine study.
LNA-based SSO for murine *Dmd* gene exon 23 skipping and its complementary oligonucleotides are shown. Sequences are shown from 5′ to 3′. Lowercase letter: DNA, uppercase letter: RNA, uppercase letter with bold and underline: LNA, **<u>C</u>**: LNA-5-methylcytosine, Toc_: tocopherol and ^: phosphorothioate.

Entry	Name	Sequence
01	LNA based SSO	a^**<u>C</u>**^c^**<u>T</u>**^c^**<u>G</u>**^g^**<u>C</u>**^t^**<u>T</u>**^a^**<u>C</u>**^c
02	Comp. RNA	G^G^U^AAGCCGA^G^G^U
03	Toc_Comp. RNA	Toc_G^G^U^AAGCCGA^G^G^U

Table S8 DNA primers used for evaluation of exon skipping efficiencies in stable cell line. DNA primers for RT-qPCR are shown. Sequences are shown from 5′ to 3′. Uppercase letters: DNA.

Entry	Gene		Sequence	Product size
01	*DMD exon 58*	Fw	AGTTCTGACCAGTGGAAGCG	156 bp
02	*skipping*	Rv	CCTCAGGAGGCAGCTCCTAT	
03	*GAPDH*	Fw	GAGTCAACGGATTTGGTCGT	185 bp
04		Rv	GACAAGCTTCCCGTTCTCAG	
05	*RPLP2*	Fw	TGGACAGCGTGGGTATCGAG	92 bp
06		Rv	CTGGGCAATGACGTCTTCAA	

FigureS1

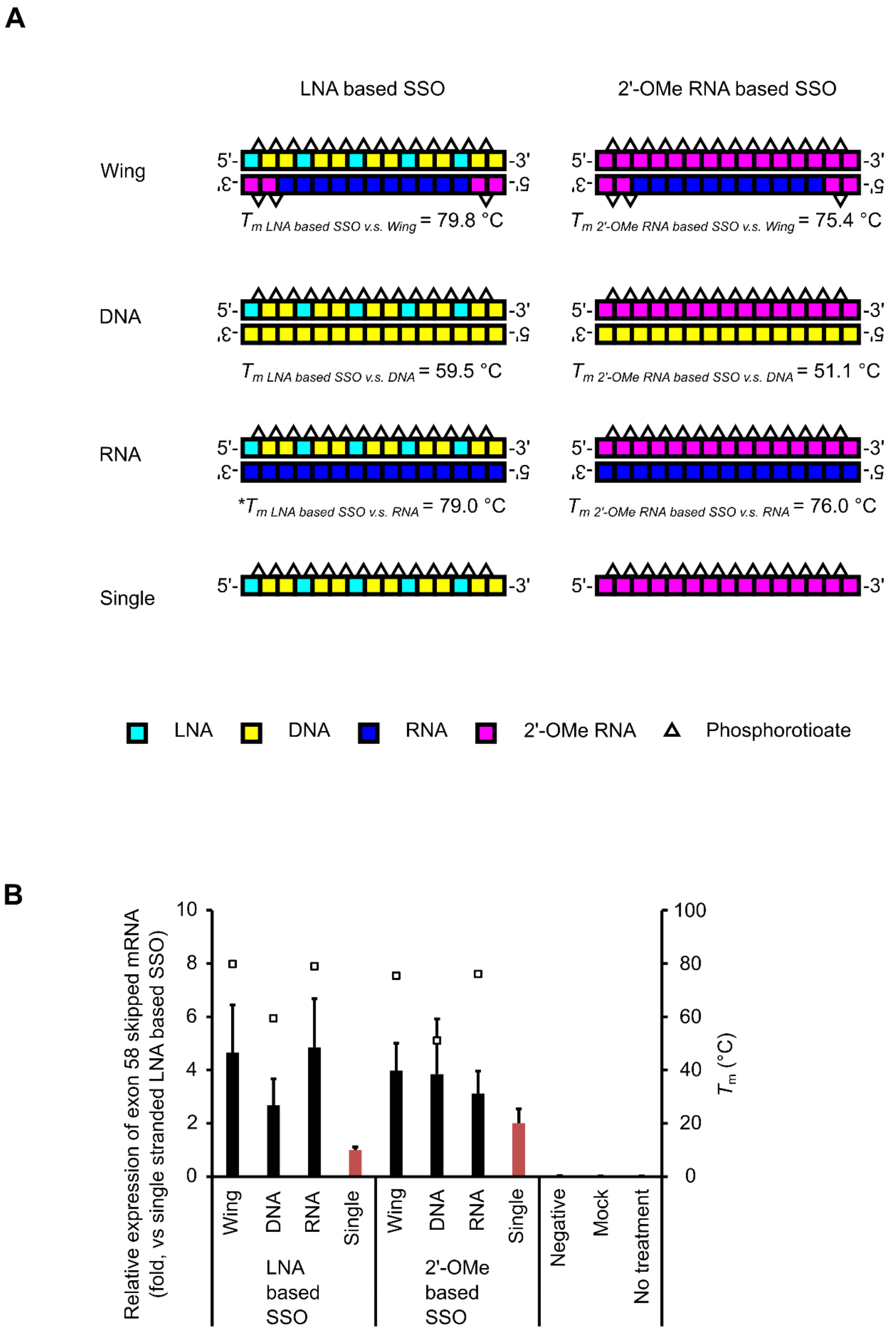

A
LNA based SSO
2'-OMe RNA based SSO
Wing
DNA
RNA
Single
5'-
-3'
3'-
-5'
$T_{m\ LNA\ based\ SSO\ v.s.\ Wing}$ = 79.8 °C
$T_{m\ 2'\text{-}OMe\ RNA\ based\ SSO\ v.s.\ Wing}$ = 75.4 °C
$T_{m\ LNA\ based\ SSO\ v.s.\ DNA}$ = 59.5 °C
$T_{m\ 2'\text{-}OMe\ RNA\ based\ SSO\ v.s.\ DNA}$ = 51.1 °C
*$T_{m\ LNA\ based\ SSO\ v.s.\ RNA}$ = 79.0 °C
$T_{m\ 2'\text{-}OMe\ RNA\ based\ SSO\ v.s.\ RNA}$ = 76.0 °C
LNA
DNA
RNA
2'-OMe RNA
Phosphorotioate
B
Relative expression of exon 58 skipped mRNA
(fold, vs single stranded LNA based SSO)
T_m (°C)
0
2
4
6
8
10
0
20
40
60
80
100
Wing
DNA
RNA
Single
Wing
DNA
RNA
Single
Negative
Mock
No treatment
LNA based SSO
2'-OMe based SSO

Figure S1 Evaluation of exon skipping activities of LNA based HDSSOs targeting 5'-site of *DMD* exon 58 in stable reporter cells.

(A) Schematic representation of the SSOs and complementary oligonucleotides used in the study. Each box shows that the one nucleotide; light blue: LNA, yellow: DNA, dark blue: RNA, red: 2'-OMe RNA. White triangles show phosphorothioate bonds. The melting temperatures of HDSSOs were also measured in at 4 μM HDSSO per 10 mM NaCl and 10 mM phosphate buffer (pH 7.2). Values represent the mean of three or four independent experiments performed. *Reference (1). (B) The result of exon skipping activities of each duplexed SSOs. The expression levels of *DMD* exon 58 skipped mRNA were measured by quantitative RT-PCR. The expression levels of *GAPDH* mRNA were used as an internal control. The graph shows the normalized *DMD* exon58 skipping activities, relative to the value in the single strand LNA based SSO-transfected cells (set at 1). Values represent the mean ± standard deviation of three independent experiments performed in duplicate. Negative; single-stranded LNA-based SSO that we reported previously as "LNA SSO (+10+24)",[1] was used as a negative control. Mock: treated with Lipofectamine RNAiMAX only; no treatment: no transfection. The white box shows the melting temperatures of HDSSOs.

FigureS2

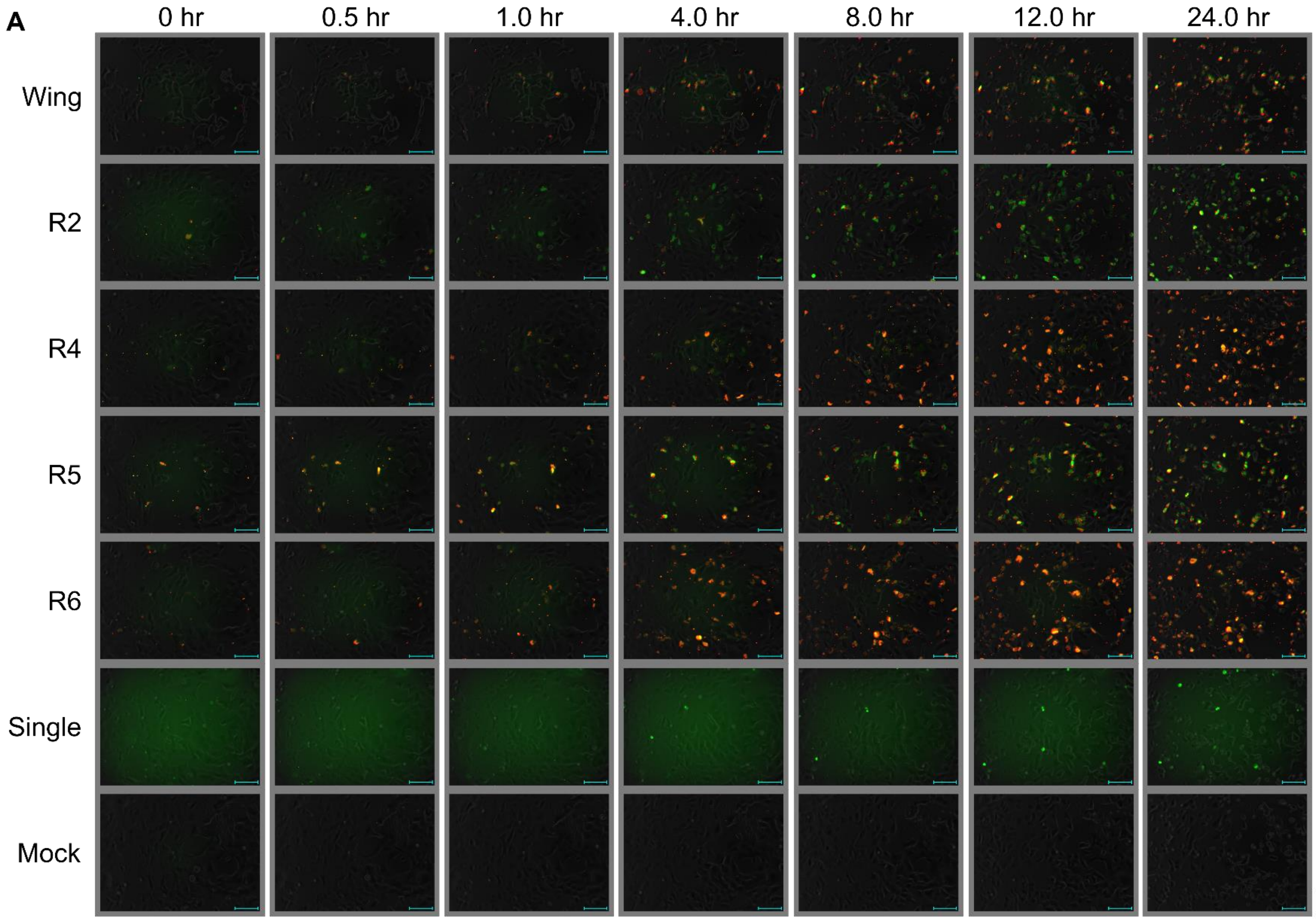
A
0 hr
0.5 hr
1.0 hr
4.0 hr
8.0 hr
12.0 hr
24.0 hr
Wing
R2
R4
R5
R6
Single
Mock

FigureS2 (continued)

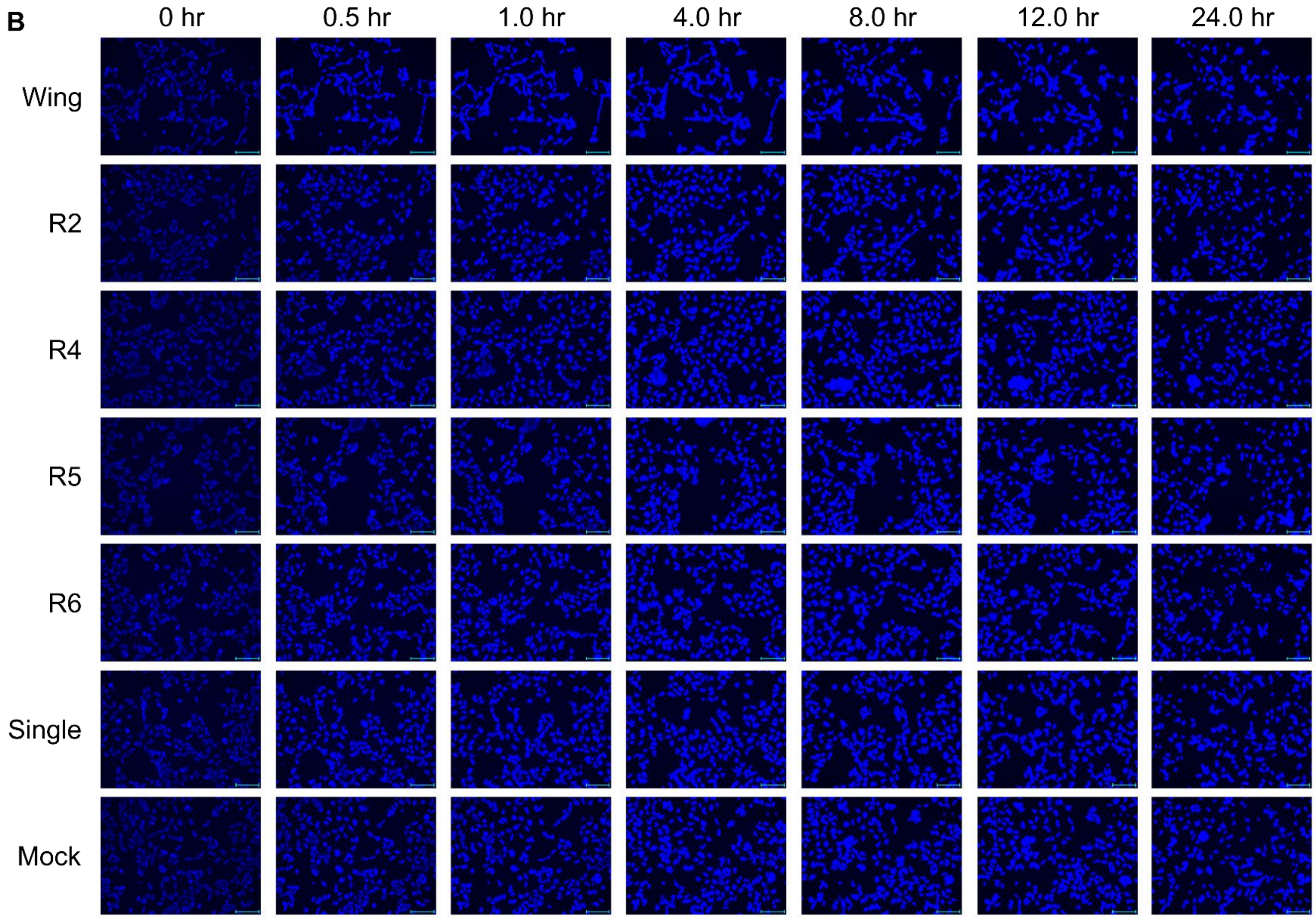

FigureS2 (continued)

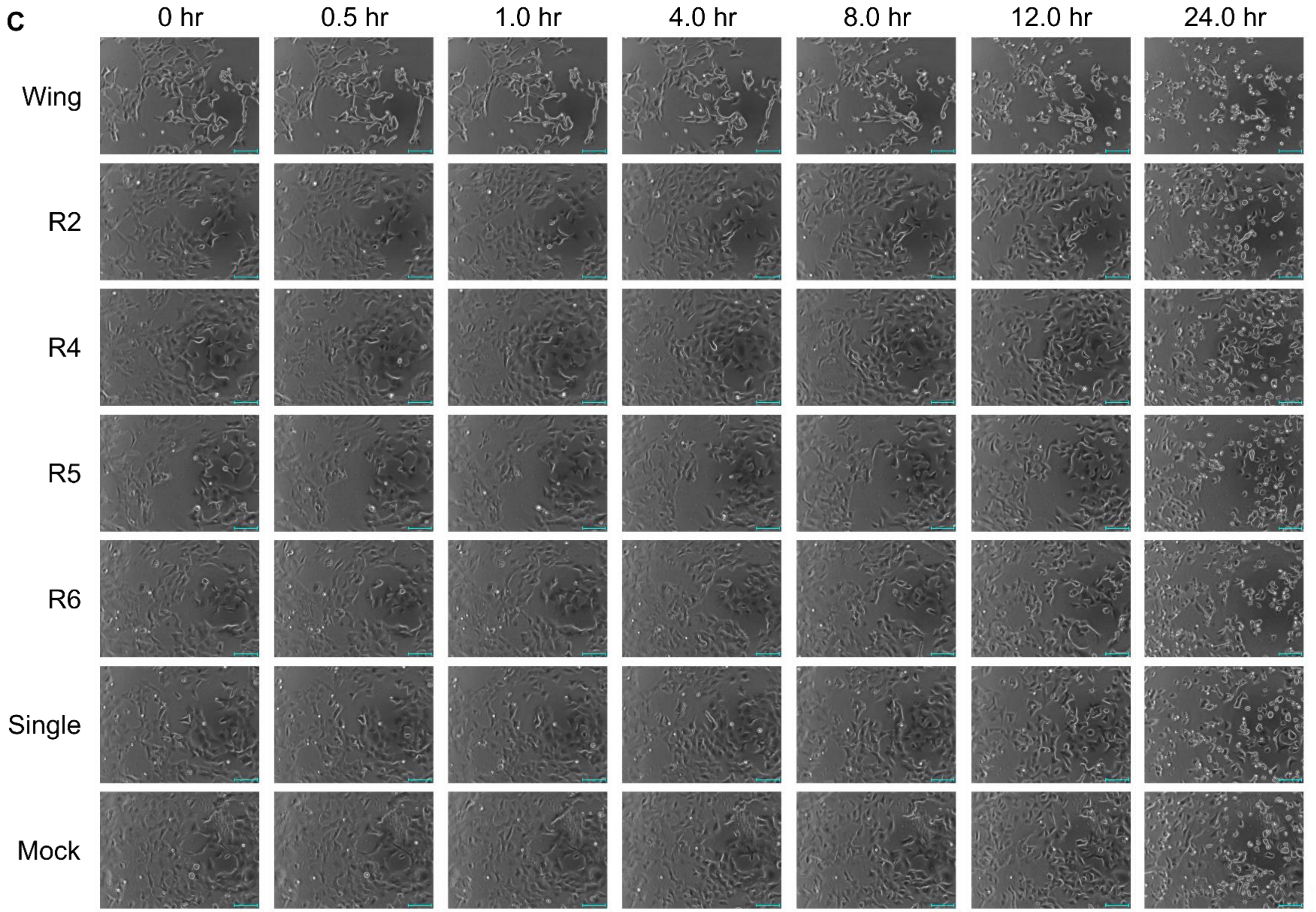

Figure S2 Time-lapse imaging of LNA based HDSSOs in HEK293 cells.

LNA based HDSSOs containing various complementary oligonucleotides were transfected into HEK293 cells using Lipofectamine RNAiMAX. Immediately after HDSSOs transfection, the images were captured with BZ-X700 (KEYENCE) at every 30 mins until 24 hr after transfection. (A-C) The captured images at 0, 0.5, 1.0, 4.0, 8.0, 12.0 and 24.0 h are shown. Green: 6-FAM conjugated SSOs, red: Alexa-647 conjugated complementary oligonucleotides, yellow: both 6-FAM conjugated SSOs and Alexa-647 conjugated complementary oligonucleotides were co-existed. Images in Figure 4 are part of this result. These images were obtained from an independent experiment performed on a different day than the experiment in both Figure 3 and Supplementary Video S1. Scale bars show 100 μm respectively. (A) The images, phase and both green and red fluorescence at same time-point, were merged using the BZ-X Analyzer software (KEYENCE). Images in Figure 4 are part of this result. (B) The images, blue fluorescence (Nuclei were stained using Hoechst 33342), are shown. (C) The images, phase contrast, are shown.

FigureS3

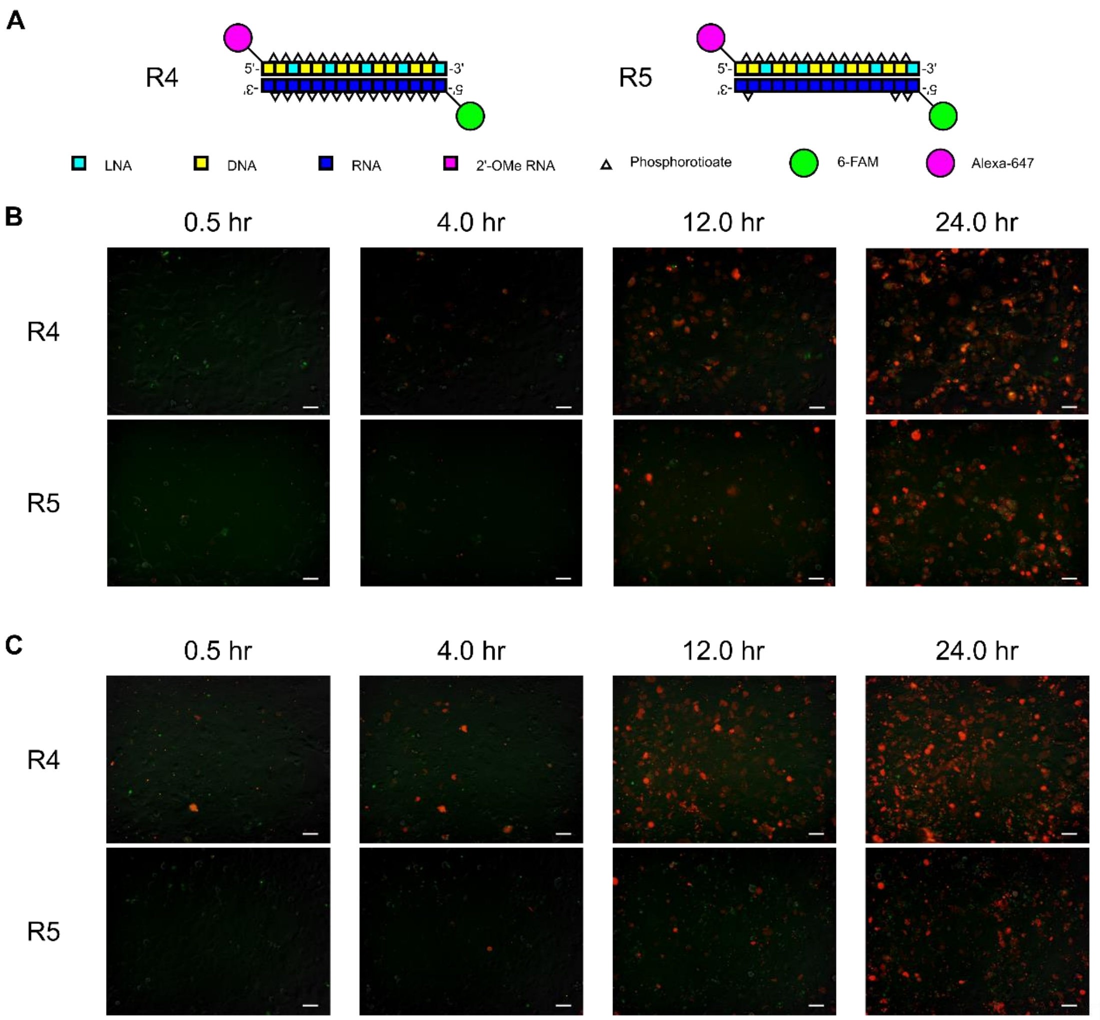

Figure S3 Time-lapse imaging of LNA-based HDSSOs in HEK293 cells with swapped fluorescent molecules.

(A) Schematic representation of the SSOs and complementary oligonucleotides used in the study. Each box shows one nucleotide; light blue: LNA, yellow: DNA, dark blue: RNA. White triangles show phosphorothioate bonds. Green: 6-FAM, red: Alexa-647. LNA-based HDSSOs containing the complementary oligonucleotides, R4 and R5, were transfected into HEK293 cells using Lipofectamine RNAiMAX. Immediately after transfection, images were captured with a BZ-X700 (KEYENCE) every 30 min until 24 h post-transfection. (B and C) The results for HDSSOs containing complementary oligonucleotides R4 and R5 are shown. Images captured at 0.5, 4.0, 12.0 and 24.0 h are shown. The images, phase, and both green and red fluorescence at the same time point were merged using BZ-X Analyzer software (KEYENCE). Red: Alexa-647 conjugated SSOs, green: 6-FAM conjugated complementary oligonucleotides; yellow: both Alexa-647 conjugated SSOs and 6-FAM conjugated complementary oligonucleotides co-existed. The

images in (B) and (C) were obtained from an independent experiment performed on a different day to show the reproducibility of results. Scale bars show 100 μm respectively.

FigureS4

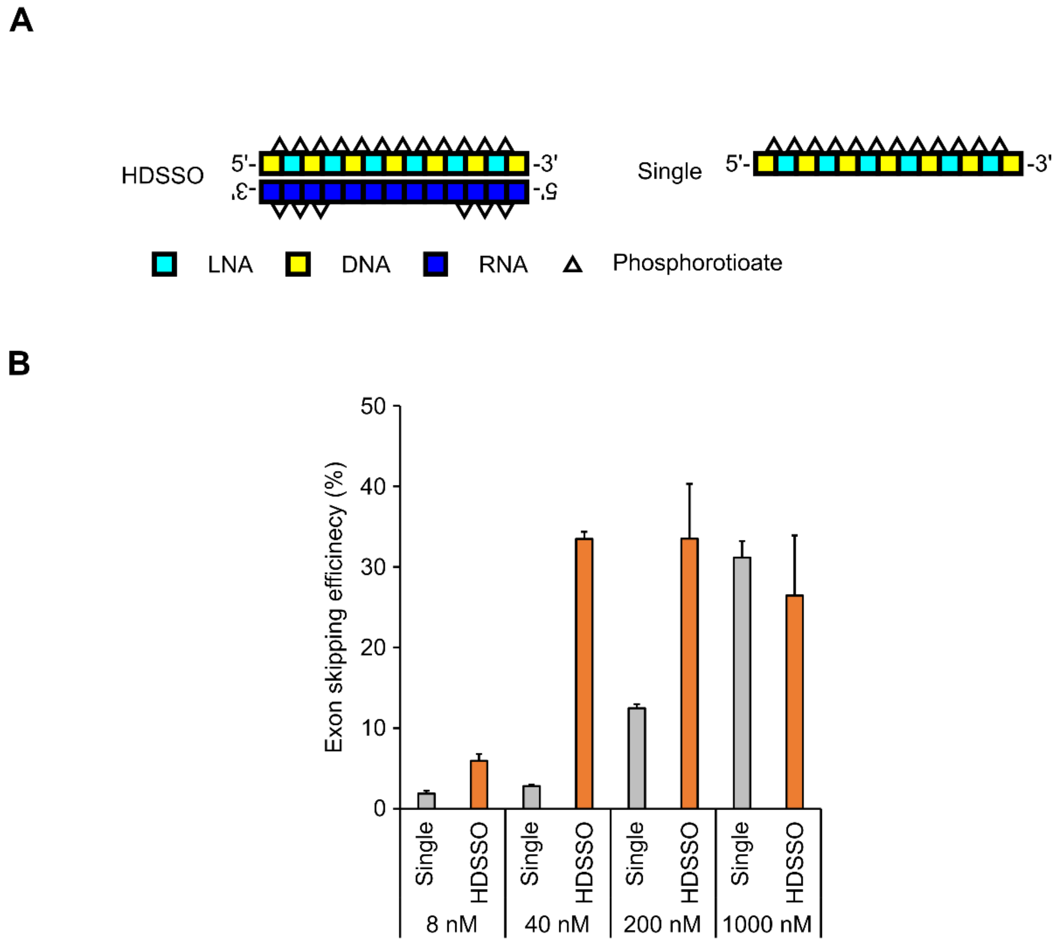

Figure S4 Evaluation of exon skipping activities of newly designed LNA based HDSSOs targeting 5'-site of *Dmd* exon 23 in C2C12 cells.

(A) Schematic representation of the SSOs and complementary oligonucleotides used in the study. Each box shows that the one nucleotide; light blue: LNA, yellow: DNA, dark blue: RNA. White triangles show phosphorothioate bonds. (B) The result of RT-PCR assay. In the 2% differentiation medium, C2C12 myotubes were incubated up to 48 hours. We then transfected Single or HDSSO (TableS7) against exon 23 of dystrophin gene at the final concentration of 8, 40, 200, or 1000 nM, respectively, with lipofectamine RNAiMAX (Thermo) and incubated them for 48 h. Then, cells were harvested. Total RNA was extracted from cells by ISOGEN 2 (NIPPON GENE) and 200 ng of total RNA was used for one-step RT-PCR (Qiagen) according to the manufacturer's instructions. The primer sequences were mEx22F 5'-ATCCAGCAGTCAGAAAGCAAA-3' and mEx24R 5'-CAGCCATCCATTTCTGTAAGG-3' for amplification from exons 22 to 24. The PCR conditions

were 50°C for 30 min and 95°C for 15 min, then 35 cycles of 94°C for 1 min, 60°C for 1 min, 72°C for 1 min, and finally 72°C for 7 min. The intensity of PCR bands was analyzed by using Bioanalayzer 2100 (Agilent), and skipping efficiency was calculated by using the following formula [(the intensity of skipped band)/(the intensity of skipped band + the intensity of unskipped band)]. Values represent the mean ± standard error of three independent experiments performed in duplicate.

FigureS5

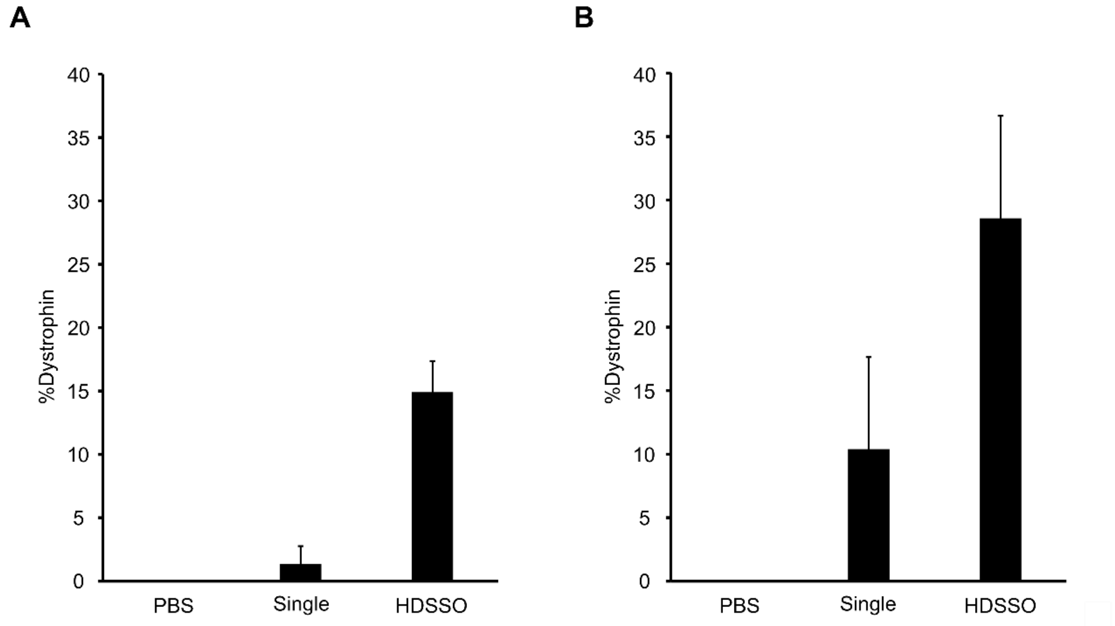

Figure S5 The exon skipping activity of LNA-based HDSSOs targeting the 5'-site of Dmd exon 23 in vivo using mdx mice.

Detection of dystrophin restoration by western blot analysis in the tibialis anterior muscle of mdx 2 weeks after intramuscular injection of SSO or HDSSO at A) 2 or B) 8 nmol compared to mdx and WT control B10 mice. The band intensities of dystrophin protein in Figure 5E of main text were detected as %dystrophin using ImageJ (fiji) software. The dystrophin expressions of WT control B10 mice was used for a calibration curve. Values represent the mean ± standard deviation (n=3 per each group).

FigureS6

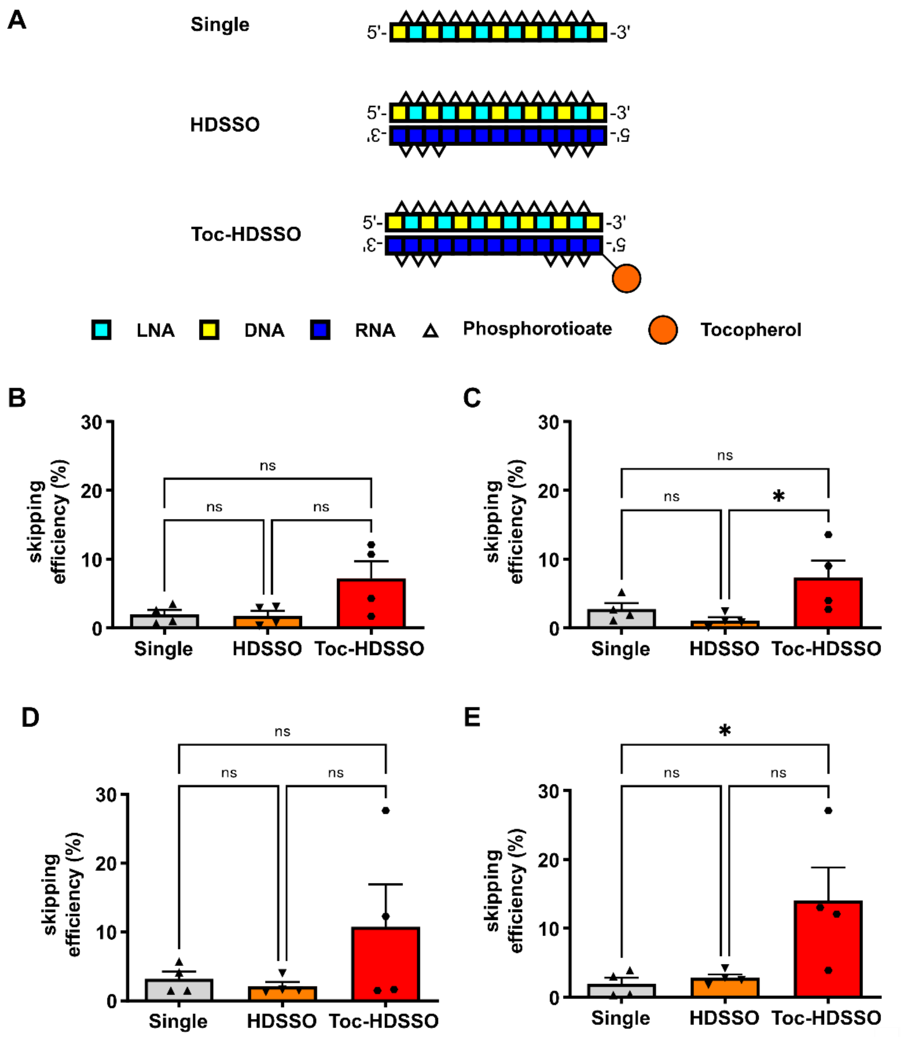

Figure S6 The exon skipping activity of LNA-based HDSSOs targeting the 5'-splice site of Dmd exon 23 in vivo using mdx mice under systemic subcutaneous injection.

(A) Schematic representation of the SSOs and complementary oligonucleotides used in the study. Each box shows one nucleotide; light blue: LNA, yellow: DNA, dark blue: RNA. White triangles show phosphorothioate bonds. (B-E) The exon skipping activity of LNA-based HDSSOs in mdx mice. Detection of exon 23–skipped dystrophin mRNA in the (B) heart, (C) diaphragm, (D) triceps and (E) quadriceps of mdx two weeks after subcutaneous injection of SSO or HDSSO at an equimolar dose 11.88 μmol/kg. Values represent the mean ± standard error (n=4 per each group). Significant differences compared to the Single were determined using Tukey's test. $*p < 0.05$.

Video S1 Merged time-lapse video of LNA-based HDSSOs in HEK293 cells.

LNA based HDSSOs containing various complementary oligonucleotides were transfected into HEK293 cells using Lipofectamine RNAiMAX. Immediately after HDSSOs transfection, the images were captured with BZ-X700 (KEYENCE) at every 30 mins until 24 hr after transfection. Green: 6-FAM conjugated SSOs, red: Alexa-647 conjugated complementary oligonucleotides, yellow: both 6-FAM conjugated SSOs and Alexa-647 conjugated complementary oligonucleotides were co-existed. The images, phase and both green and red fluorescence at same time-point, were merged using the BZ-X Analyzer software (KEYENCE). The time-lapse videos of merged images at every 30 mins are shown. Images in Figure 3 are part of this result. These images were obtained from an independent experiment performed on a different day than the experiment in both Figure 4 and Figures S2A-C. (A-E) Merged time-lapse video, HEK293 cells transfected with HDSSOs containing (A) Wing, (B) R2, (C) R4, (D) R5 and (E) R6 respectively, are shown. (F) Merged time-lapse video, HEK293 cells transfected with Single (single LNA modified SSOs), is shown. (G) Merged time-lapse video, HEK293 cells treated with Lipofectamine RNAiMAX only (mock), is shown. Scale bars show 100 μm respectively.

Reference

1. Shimo, T., Tachibana, K., Saito, K., Yoshida, T., Tomita, E., Waki, R., Yamamoto, T., Doi, T., Inoue, T., Kawakami, J. *et al.* (2014) Design and evaluation of locked nucleic acid-based splice-switching oligonucleotides in vitro. *Nucleic Acids Res*, **42**, 8174-8187.
